# Multi-class, unsupervised detection and classification of biological and anthropogenic sounds in coral reefs

**DOI:** 10.1371/journal.pcbi.1014516

**Published:** 2026-07-20

**Authors:** Daniel Duane, Matthew T. Duggan, Erika Berlik, Marc S. Dantzker, Aaron N. Rice, Lauren A. Freeman

**Affiliations:** 1 Naval Undersea Warfare Center, Newport, Rhode Island, United States of America; 2 Cornell K. Lisa Yang Center for Conservation Bioacoustics, Cornell University, Ithaca, New York, United States of America; 3 Cornell Lab of Ornithology, Cornell University, Ithaca, New York, United States of America; 4 FishEye Collaborative, Arlington, Virginia, United States of America; Technological University Dublin, IRELAND

## Abstract

Analyzing the complex and diverse soundscapes of ecosystems such as coral reefs remains a challenge for understanding environmental dynamics and processes. While machine learning techniques can significantly improve detection and classification capabilities, applications of traditional supervised learning to underwater acoustics are limited by the size and class-coverage of labeled datasets. Unsupervised machine learning offers the potential to detect and classify sounds without the guidance of human labels, including signals that were unknown to the human analyst. However, the majority of previously developed unsupervised approaches characterize reef soundscapes from correlative metrics without identifying specific sounds, and the few that detect individual signals have been trained on limited data (<10 days), which constrains the potential to generalize across datasets and geographical localities. Here, a convolutional autoencoder was built and trained on year-long acoustic datasets from four Hawaiian coral reefs, and latent embeddings were clustered using Gaussian mixture modeling. A total of 29 classes were automatically generated, and a manual review of samples in each class determined that nine of the classes corresponded to distinct biological and anthropogenic sounds. The classes were identified to be two call types from the damselfish, *Dascyllus albisella*, parrotfish feeding sounds, holocentrid calls, an unidentified fish sound, three humpback whale song units, and ship noise. The classifier was found to be robust against an independently-collected test dataset with *D. albisella* calls (AUC = 0.9) with no extra training on the labels. Diel, lunar, and seasonal trends were observed for all nine classes, including previously-unidentified responses of the holocentrid and unknown fish groups to lunar illumination. This work demonstrates the capability of unsupervised algorithms to cluster acoustic signals into identifiable biological and anthropogenic categories in order to examine and characterize ecological trends.

## Author’s summary

Detection and classification of biological sounds is essential for monitoring the restoration progress of coral reefs, yet most artificial intelligence (AI) methods require large, hand‑labeled training datasets, which are difficult to obtain in acoustically complex reef environments. Here we present an unsupervised AI algorithm that automatically detects and clusters sounds from acoustic recordings with no manual annotation. We applied the algorithm to four concurrent, year‑long recordings from Hawaiian coral reefs, and it automatically grouped detections into nine distinct signal types: two call types from Domino damselfish, feeding sounds from parrotfish, calls from holocentrids (squirrelfishes and soldierfishes), an unidentified fish sound, three separate humpback whale song units, and ship noise. When tested on an independent dataset with labeled Domino damselfish calls, the model correctly identified more than 82% of damselfish calls while producing false positives on fewer than 15% of non‑damselfish sounds. All nine classes exhibited patterns linked to sunlight or the seasons, and we discovered previously‑unreported responses of the holocentrid and unknown fish groups to moonrise and lunar phase. This work demonstrates that unsupervised AI can detect and cluster sound types and uncover ecological trends without the guidance of a human analyst.

## 1. Introduction

Coral reefs are acoustically dynamic environments, with hundreds of diverse biological sounds often contained within a single minute of acoustic data [e.g., 1,2] alongside physical and anthropogenic noise [3,4]. These soundscapes serve as important indicators of reef health, with acoustic diversity and activity shown to increase recruitment of pelagic fish, coral, and crustacean larvae [5–11], and to directly correlate with more resilient reefs [5,12,13]. However, to date, reef ecosystem function as inferred through soundscape metrics has largely been correlative, based on unidentified sound classes [12,14,15], band-limited sound levels [4,16–19], or acoustic indices [5,20–24], all of which lack taxonomic identification. By parsing multiple individual biological sounds and identifying them to a more precise taxonomic level, coral reef soundscapes can be interpreted to provide improved ecological context and actionable metrics for reef monitoring, conservation, and restoration. This is critical because natural resource management is primarily focused on individual species rather than sound production [2].

The application of machine learning detectors and classifiers allows for the identification of individual signals that would be missed by traditional intensity-based metrics and acoustic indices. For automated detection of fish sounds, supervised machine learning has been typically applied to large, labeled datasets focusing on a single call type, including calls from groupers [25,26], damselfishes [27], and toadfishes [28]. While effective for identifying spatial or temporal call patterns, these techniques are typically not transferable to other environments/locations, other sensors, or other call-types. Other supervised detectors have been trained on fish calls more broadly, without differentiating call-type or species [14,15].

In contrast to supervised learning, unsupervised techniques enable rapid and inexpensive signal labeling without manual annotation. Unsupervised learning reduces inter- and intra-observer variability in event classification by circumventing subjective human interpretation, which can be particularly useful for acoustically diverse datasets where individual sounds are unknown or difficult to disentangle. Previous unsupervised algorithms have been trained to identify biological choruses in long-term spectral averages with minute-scale temporal resolution [29–33], or to characterize soundscape recordings without identifying individual signals [23,24]. While a number of studies have used unsupervised clustering to detect and identify sounds associated with marine mammals [34–39], fewer studies have used unsupervised techniques to classify individual fish sounds. In a study by Noble et al. [40], handpicked spectral and temporal features were extracted from manually labeled fish calls in order to cluster them into 55 unidentified sub-groups. A study by Ozanich et al. [41] differentiated fish and whale vocalizations using two unsupervised approaches: 1) clustering of handpicked spectral and temporal features similar to Noble et al. [40], and 2) clustering of deep latent features generated by convolutional autoencoders, where the deep learning approach was found to yield significant improvements in classification accuracy. The algorithms developed by Noble et al. [40] and Ozanich et al. [41] only provided broad categorical labels and were both applied to less than ten days of data, which constrained the ability to interpret soundscape composition and resolve diel or seasonal ecological trends.

Here, an unsupervised deep clustering algorithm was trained on four concurrent, year-long acoustic datasets from Hawaiian coral reefs, using an autoencoder-Gaussian mixture model framework similar to Ozanich et al. [41]. A simple manual review of automatically-generated clusters identified nine classes corresponding to distinct acoustic signals, including two call types from the Domino damselfish, *Dascyllus albisella*, soniferous signatures from holocentrids (squirrelfishes and soldierfishes), an unidentified fish call, scraping from parrotfish grazing, three song units from humpback whales, and noise from ship traffic. The clustering algorithm was verified against an independently collected, human-annotated dataset containing labeled *D. albisella* calls with synchronized video-audio array verification (AUC = 0.9). Seasonal and diel characteristics of well-studied signals (such as humpback whale and damselfish sounds) are consistent with known behaviors, while the temporal patterns of less-studied signals reveal previously-unidentified ecological trends, including the response of the holocentrid group to lunar illumination. This work demonstrates the capability of unsupervised algorithms to derive biologically meaningful acoustic classes from unlabeled reef soundscapes at an event-level resolution and provide ecological insights through long-term analysis of species- and family-level acoustic signatures.

## 2. Methods

**Fig 1:**
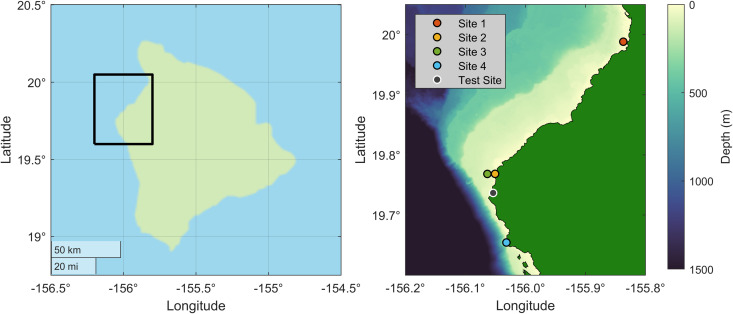
Map of survey sites on the western coast of Hawai’i Island. Red, yellow, green, and blue dots correspond to long-term single-sensor survey sites, and the black dot corresponds to the test site with a synchronized video-audio array. Bathymetric data is from the Main Hawaiian Islands Multibeam Bathymetry Synthesis (https://www.soest.hawaii.edu/hmrg/multibeam).

Long-term passive acoustic measurements were collected at four survey sites with sensor depths ranging from 20-30 m off the western coast of Hawai’i Island (Fig 1). Survey sites were selected based on prior local knowledge to capture a wide range of coastal reef settings. HTI-96-min hydrophones were deployed with Loggerhead LS1 (Survey Sites 1 and 4) or Loggerhead LS1X (Survey Sites 2 and 3) recording packages. Acoustic recorders were set to record at a sampling rate of 96 kHz, recording for 1 minute every 15 minutes for the LS1 recorders and 1 minute every 10 minutes for the LS1X recorders to accommodate the 1-year deployment duration. The sensitivity of the HTI 96-min hydrophones used here was -170 dB re V/μPa, and the frequency range was 2 Hz to 30 kHz. A synchronized video-audio array was independently deployed at a fifth location (“Test Site”, Fig 1) in order to provide ground-truth verification of calls from *Dascyllus albisella* (Domino damselfish). This is a well-documented vocalizing species in Hawaiian coral reefs [42–45], but few of their sounds are publicly available for model training. This passive acoustic camera combines an Insta-360 X2 360° camera within a tetrahedral hydrophone array and is described further in Dantzker et al [2]. The passive acoustic camera was deployed near nests of *Dascyllus albisella* and allowed for the opportunity to match sounds from specific focal fish species and their associated behaviors with vocalizations.

The data analyzed from the four survey sites spans from May 1, 2020 at 00:00 local time to May 1, 2021 at 00:00 local time. Each one-minute audio sample was downsampled to a sampling frequency of 1600 Hz and run through a 4^th^-order highpass Butterworth filter with critical frequency 30 Hz to reduce low-frequency seismic noise. Spectrograms were generated with 32-sample fast-Fourier transforms with an overlap of 28. Frequencies between 150 and 750 Hz were isolated in order to target a frequency band of interest where diverse biological sounds are present [46].

Detections were made using the Wang & Willett power-law detector, which was chosen for its suitability for transient signals of unknown structure. The power law statistic PL(t) is defined [47] as


$$\begin{array}{c} PL(t)=\;\int_{f}{\left(\frac{P_{xx}(t,f)}{B(t,f)}\right)}^{\nu }df \end{array}$$ (1)

where $P_{xx}(t,f)$ is the power spectral density in linear scale at time t and frequency f, B(t,f) is the background noise level, and ν=1.7 [47]. Here B(t,f) was calculated for each frequency band as the 3-second median of $P_{xx}(t,f)$ centered at time t. The power law statistic was then smoothed using a time-domain Gaussian filter with a standard deviation of 10. This filter width was chosen to focus the detector on longer-duration sounds (on the order of the 0.36 s sample window used in subsequent analysis) and to help ensure detections remained centered within the sample window. Peaks were then identified at a threshold of one standard deviation above the one-minute mean of the filtered output. This low detection threshold was intentionally chosen so that the automated clustering algorithm would be trained to distinguish relevant signals from background or low signal to noise ratio (SNR) samples. For each detection, a spectrogram sample (${PSD}_{sample}$) centered at the detection time was generated with size 12x144, corresponding to frequency range 150–750 Hz and duration 0.36 s. Spectrogram samples were standardized according to


$$\begin{array}{c} PSD^{\prime }=\frac{{PSD}_{sample-}\mu_{sample}}{\sigma_{sample}} \end{array}$$ (2)

where $\mu_{sample}$ and $\sigma_{sample}$ are the mean and standard deviation of ${PSD}_{sample}$ across both time and frequency bins. This standardization step ensures that signals with similar temporal/spectral characteristics but different intensities will be clustered similarly. Normalized samples were then obtained by clipping $PSD^{\prime }$ according to


$$\begin{array}{c} {PSD}_{norm}=\left\{ \begin{array}{cc} \text{1} & PSD^{\prime }>\text{2}\\ PSD^{\prime }-\text{1} & \text{1}<PSD^{\prime }\leq \text{2}\\ \text{1} & PSD^{\prime }\leq \text{0} \end{array}\right. \end{array}$$ (3)

effectively setting the lower threshold at one standard deviation above the within-sample mean and the upper threshold at two standard deviations above the within-sample mean. Samples were clipped in this way in order to zero out background fluctuations and emphasize higher-SNR features. The detector was run on the full year of data in all four survey sites. In order to equalize detection rates between sites with different duty cycles, we discarded every third detection at Survey Sites 2 and 3, which had 1.5 times greater temporal coverage than Sites 1 and 4.

**Fig 2:**
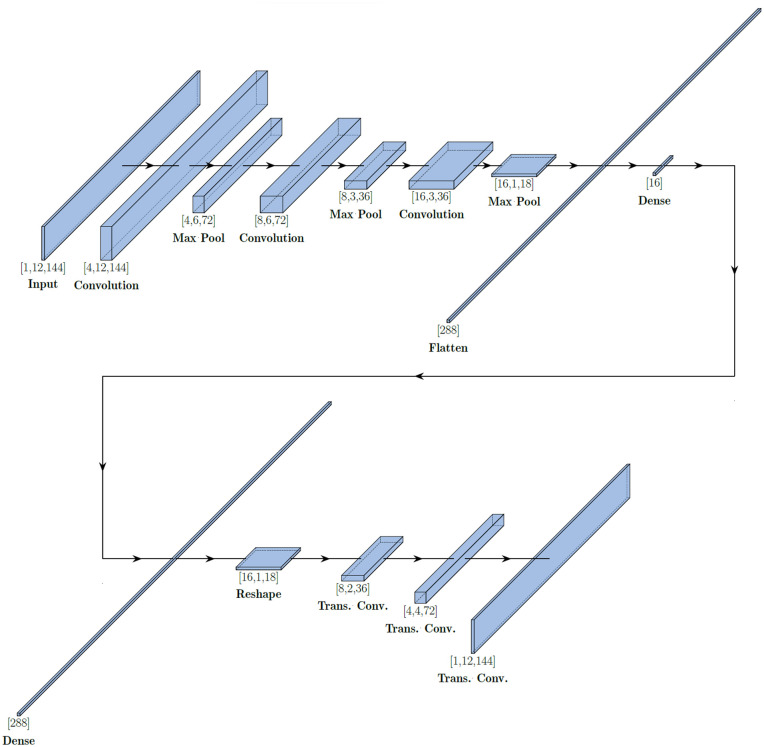
Architecture of the convolutional autoencoder. The encoder compresses a 12x144 input into a 16 element latent embedding (top row). The decoder constructs a recreation of the input using only the latent embedding (bottom row).

**Fig 3:**
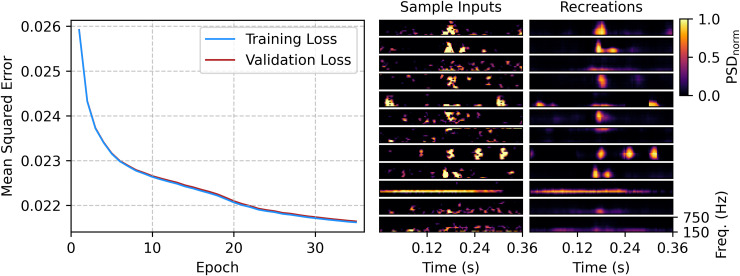
Training/validation loss and sample recreation. Training and validation loss (left) are nearly identical after training ends, indicating that the autoencoder is not overfitting to the training set. Randomly-selected sample input spectrograms (middle) and reconstructions (right) show the autoencoder is retaining the basic spectral and temporal features in the latent embeddings.

A total of 7,767,943 detections were made across the four survey sites (1,514,293 in Site 1, 1,933,200 in Site 2, 1,931,952 in Site 3, and 2,388,498 in Site 4). The detections were randomly split into a training set (90%) and a held-out validation set (10%) and then fed into a convolutional autoencoder which compresses inputs into a 16-dimensional latent space before reconstruction (Fig 2, Table 1). This dimensionality was determined by testing several configurations, selecting the smallest latent space that ensured the reconstructed outputs captured the essential spectral and temporal features of the input spectrograms. Training utilized the Adam optimizer with a learning rate of 0.0001 and a batch size of 64, minimizing the mean squared error (MSE) between input and output tensors. Training concluded automatically when the epoch-to-epoch MSE reduction fell below 1 × 10 ⁻ ⁵, requiring 35 epochs to reach convergence on the training set (Fig 3). On a machine equipped with 128 GB of RAM and two NVIDIA TITAN V GPUs, this training process was completed in less than 26 hours.

**Table 1:** Autoencoder architecture, ReLu = Rectified linear unit.

Layer	Input shape	Output shape	Kernel size	Stride	Padding	Filter	Activation
Convolution	[1,12,144]	[4,12,144]	[3,3]	[1,1]	[1,1]	4	ReLu
Max Pool	[4,12,144]	[4,6,72]	[2,2]	[2,2]	[0,0]	--	--
Convolution	[4,6,72]	[8,6,72]	[3,3]	[1,1]	[1,1]	8	ReLu
Max Pool	[8,6,72]	[8,3,36]	[2,2]	[2,2]	[0,0]	--	--
Convolution	[8,3,36]	[16,3,36]	[3,3]	[1,1]	[1,1]	16	ReLu
Max Pool	[16,3,36]	[16,1,18]	[2,2]	[2,2]	[0,0]	--	--
Reshape	[16,1,18]	[1,1,288]	--	--	--	--	--
Fully Connected	[1,1,288]	[1,1,16]	--	--	--	--	ReLu
Fully Connected	[1,1,16]	[1,1,288]	--	--	--	--	ReLu
Reshape	[1,1,288]	[16,1,18]	--	--	--	--	--
Trans. Conv.	[16,1,18]	[8,2,36]	[2,2]	[2,2]	[0,0]	16	ReLu
Trans. Conv.	[8,2,36]	[4,4,72]	[2,2]	[2,2]	[0,0]	8	ReLu
Trans. Conv.	[4,4,72]	[1,12,144]	[3,2]	[3,2]	[0,0]	4	ReLu

After training, the encoder reprocessed the entirety of the dataset to extract latent feature representations. These representations were clustered using Gaussian mixture models (GMMs) with k-means++ initialization and full covariance matrices in order to account for potential correlations between latent features. To ensure clustering stability and mitigate the risk of the expectation-maximization algorithm converging to suboptimal local extrema, we employed a standard multi-start protocol, selecting the final model based on the maximum log-likelihood achieved across ten random restarts. To assess sensitivity to the number of clusters (k), we computed standard model-selection metrics including the Akaike Information Criterion (AIC), Bayesian Information Criterion (BIC), Silhouette score, and Calinski–Harabasz index, across candidate cluster counts ranging from 10 to 45. We observed that these traditional metrics for evaluating cluster count did not converge to an optimal k for this dataset (S1 Fig). This is likely a consequence of a high proportion of noisy or ambiguous samples, which are prevalent in complex coral reef soundscapes. Therefore, we focused our model selection on the portions of the data that were well-modeled by the GMM. For each candidate cluster count k, we identified “well-defined” clusters—those where >10% of assigned samples had likelihoods >0.99—and computed the Akaike Information Criterion exclusively using samples within these well-defined clusters. This approach evaluated the model’s ability to describe well-structured data, while remaining robust to poorly-fit, noisy outliers. The optimal model (k = 29) minimized this modified AIC.

To interpret the acoustic content of these unsupervised groupings, we manually inspected 100 high-likelihood samples (likelihood L > 0.99) per cluster by: 1) viewing their normalized spectrograms, and 2) listening to their raw audio. This manual review step is necessary to assign biological and ecological meaning to the automatically generated clusters. We focused this review on high-likelihood samples to establish the dominant acoustic signatures of each soft cluster, acknowledging that low-likelihood samples at the cluster peripheries would likely contain more ambiguous sounds or noise, a natural consequence of the complex acoustic environment. When possible, clusters were associated with source-specific labels (e.g., species or vessel) based on sounds documented in the literature [e.g., 46, 48, 49] or focal observations confirmed with video [2].

## 3. Results

**Fig 4:**
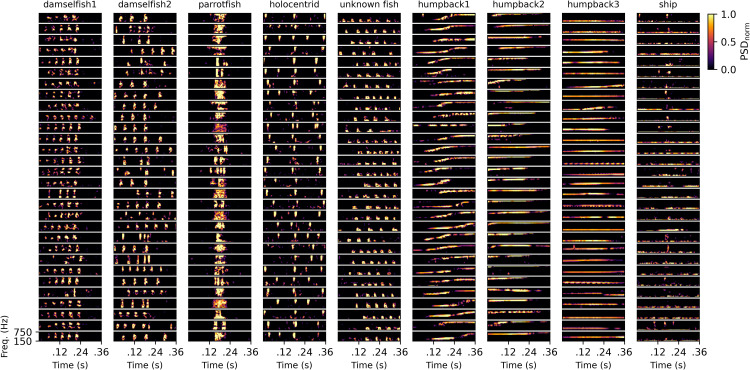
Randomly selected spectrograms from high-likelihood (L>0.999) samples for each of the 9 automatically-generated classes. Audio files containing sample sounds from each class are in S1 Audio.

Manual inspection of automatically generated clusters revealed that nine of the 29 clusters were associated with identifiable manmade and biological signals. These included two distinct sounds produced by *Dascyllus albisella* (“damselfish1,2”), scraping sounds from parrotfish feeding on coral (“parrotfish”), sounds from holocentrids (“holocentrid”), an unidentified fish sound (“unknown fish”), three distinct humpback whale song units (“humpback1,2,3”), and ship noise (“ship”). Sounds from holocentrid fishes were inferred based on the similarity to sounds produced by *Myripristis berndti* and other members of the family [50–52], but we did not feel confident about assigning species-level labels to the holocentrid class. While the identified humpback song unit clusters represent acoustically distinct signals, we did not assess whether they are functionally distinct within the context of songs they are part of. Example spectrograms from very high-likelihood (L>0.999) samples in each of the characterized classes are shown in Fig 4, and example spectrograms with variable thresholds for inclusion (L > 0.9, 0.5, 0.25, and 0) are shown in S2 Fig. While consistent spectral and temporal features are generally preserved as the detection threshold is lowered, this adjustment leads to the inclusion of noisy or misclassified samples.

Spectrogram samples for the uncharacterized clusters (numbered 1–20) are shown in S3 Fig. Manual review confirmed that many of these uncharacterized clusters contain a mixture of unidentified biological pulses and non-biological transient sounds, or low-SNR signals with few visible features in the spectrograms. One of these uncharacterized clusters (“18”) was found to contain a mixture of humpback whale sounds and ship tonals, indicating some confusion between these signal types.

To evaluate the statistical properties of the 29 clusters automatically generated by the Gaussian mixture model, we analyzed their internal variance, inter-cluster similarity, and classification confidence. The generalized variance for each cluster is shown in S4 Fig, where the comparatively high variance of the damselfish2 and holocentrid classes suggests they encompass a diverse range of vocalizations, and the lower variance of the parrotfish class indicates a narrower range of sound types. The probability distributions for these clusters were found to be mathematically distinct, as the Bhattacharyya coefficients for nearly all cluster pairs were below 0.36, indicating minimal overlap (S5 Fig). An analysis of likelihood scores revealed that the nine characterized sound classes are dominated by high-confidence detections, with prominent modes above a 0.99 likelihood (S6 Fig). In contrast, many of the uncharacterized clusters lack a high-confidence peak, suggesting ambiguous or noisy samples with uncertain assignments.

**Fig 5:**
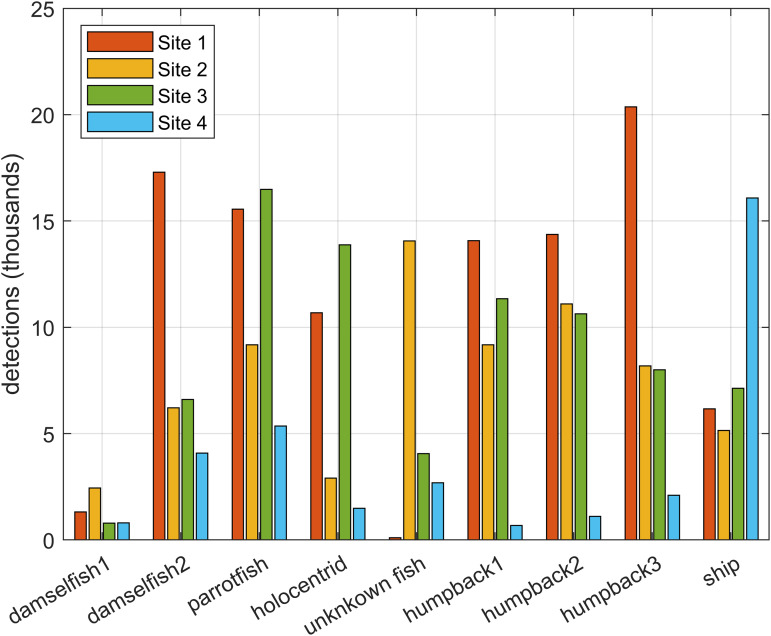
Number of detections in each class (L>0.99) across the 12 month recording period for the four survey sites.

The distribution of detections for the characterized classes across all survey sites is shown in Fig 5. Significant variations in detection rates for each class are seen across the survey sites. Survey Site 1 saw a majority of damselfish2 call detections (51%) and a plurality of detections across all three humpback classes (40%, 39%, and 52%, respectively), as well as a strikingly low number of unknown fish detections (<0.5%). Site 2 saw a majority of unknown fish detections (67%) and a plurality of damselfish1 detections (46%). Site 3 saw a plurality of holocentrid (48%) and parrotfish (35%) detections. Site 4 was dominated by anthropogenic noise (47% of ship detections) with comparatively few biological detections (3.4% of humpback detections, 5.1% of holocentrid detections, and <15% for all other biological classes). The number of detections per class ranged from 20,000–50,000, with the exception of the damselfish1 class, where there were approximately 5,000.

**Fig 6:**
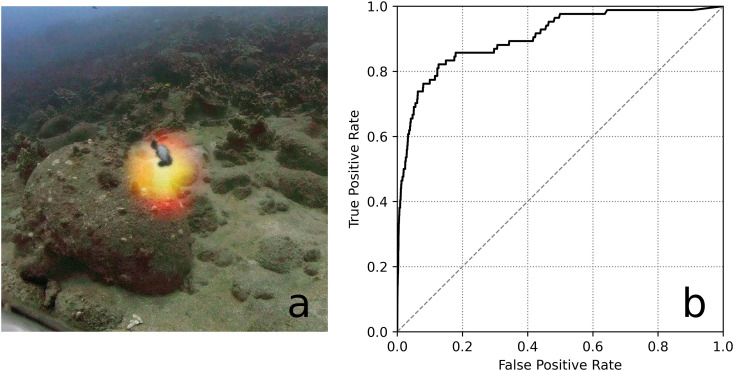
Evaluating cluster assignments with an independent, labeled dataset. Detection performance for the damselfish1 and 2 classes was evaluated using a 1-hour test dataset where a collocated acoustic array and 360° video system allowed for the visual identification of vocalizing fish. A sample video frame is shown in **(a)**, with an overlain visualization of the sound field energy distribution corresponding to the position of a *Dascyllus albisella*. A Receiver Operating Characteristic (ROC) curve for damselfish detections is shown in **(b)**, with an area under the curve of 0.9. The visualization of the sound field energy is masked to make the individual visible in this illustration.

Detection performance for the damselfish1 and 2 classes was evaluated using the labeled dataset of verified *Dascyllus albisella* calls collected at the Test Site. The passive acoustic camera was deployed near a nest of *Dascyllus albisella* on July 27, 2022, and manual detections were made over a total of 57 minutes spanning 11:32–11:59 and 12:57–13:27 local time. 85 manual labels of the *Dascyllus* calls were verified by collocating beamformed acoustic detections with images of the individual from the 360° video imagery (Fig 6a). All of these sounds were associated with *Dascyllus* courtship/territorial displays, and video samples for four of the calls can be found at https://www.fisheyecollaborative.org/fish-sounds/dascyllus-albisella. The entire 57-minute dataset was run through the detection mechanism outlined in Section 2, where 6,098 initial detections were made, 82 of which overlapped with the manual labels. The 6,098 detections were classified using the pre-trained encoder and GMM clustering algorithms, with no extra training on the labeled dataset. Detections were classified as damselfish when $L_{\text{1}}+L_{\text{2}}>T$ where $L_{\text{1}}$ and $L_{\text{2}}$ are the likelihood scores for the damselfish1 and damselfish2 classes assigned by the Gaussian mixture model, and T is an adjustable detection threshold. A ROC curve is generated by varying T from 0 to 1 (Fig 6b). True positive rates of >82.5% are possible with false positive rates of <15%, and the area under the curve is 0.90. To validate the choice of this clustering framework, we also implemented standard k-means clustering using the same learned latent representations to serve as a baseline comparison (S7 Fig). The Gaussian mixture model demonstrated improved discrimination performance relative to k-means, achieving a higher ROC AUC (0.90 vs. 0.85) and Precision-Recall AUC (0.32 vs. 0.23). This supports the use of GMM clustering for this application.

**Fig 7:**
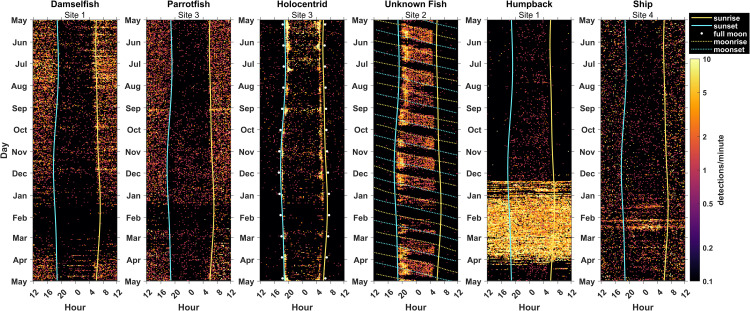
Unique diel, lunar, or seasonal trends are observed for all classes. Detection rates are shown as a function of time of year (vertical axis) and time of day (horizontal axis) for classes at selected survey sites, where the two damselfish call types and the three humpback song units were merged into singular classes. Solid yellow and cyan lines respectively correspond to sunrise and sunset, dashed yellow and cyan lines respectively correspond to moonrise and moonset, and white dots correspond to full moon. Detections were made here using a likelihood threshold of inclusion L > 0.99. Recreations of these plots with variable thresholds (L > 0.9, 0.5, 0.25, and 0) are shown in S8 Fig. Similar plots for all survey sites are shown in S9 Fig.

The diel and seasonal characteristics of the signals in each class are visualized in Fig 7. For classes where diel and seasonal characteristics are known, temporal patterns are consistent with previous observations. Humpback whale vocalizations occurred almost exclusively between the months of December and April, consistent with the known migration patterns of humpback whales to Hawaiian breeding grounds during the winter [53,54]. Damselfish calls and parrotfish scrapes occurred during the day, consistent with previous acoustic observations [27,55]. Ship noise occurred at sporadic intervals mostly during daytime hours, which can be explained by the prevalence of recreational vessels associated with day trips rather than cargo vessels or cruise ships at these survey sites. Significant increases in “ship” detections occurred during the nights of February 5 and 15, and manual inspection of samples confirmed that increased seismic activity that bled into frequencies above 150 Hz was misclassified here as ship noise.

The output of the classifier also yielded insight into the temporal patterns of signals that have not been extensively studied. In particular, the holocentrid and unidentified fish calls displayed strong diel and lunar patterns. The unknown fish call occurred almost exclusively at night, during hours where the moon was not in the sky (dashed cyan and yellow lines, Fig 7). While changes in sound pressure level associated with moonrise have indicated a biological response to moonlight in this dataset [17], the automated detector and classifier allowed us to isolate the specific signals responsible for the lunar chorus. The holocentrid calls were most prominent in the 1–2 hours after sunset and before sunrise, with increases in activity during the full moon (white dots, Fig 7).

## 4. Discussion and conclusion

We demonstrated the ability to automatically detect and cluster individual reef sounds with no manual labels, advancing event-level signal detection by deploying established unsupervised learning architectures across multi-site, long-term soundscapes. After training on four concurrent, year-long datasets from Hawaiian coral reefs, our approach successfully partitioned sounds into distinct acoustic groups, which were then manually interpreted to identify ecologically meaningful categories, including five distinct fish call classes, three humpback whale song units, and a ship noise class. The diel and seasonal patterns of damselfish, parrotfish, and humpback whale detections are consistent with previous observations, with damselfish and parrotfish active in the daytime [27,55] and humpback songs detected in the winter months [53]. The holocentrid and unknown fish signals have not been identified previously, likely because they occur during the nighttime when diver or camera surveys are not viable. The holocentrid and unknown signals exhibit distinct responses to sunrise, sunset, and lunar illumination, demonstrating the capability of unsupervised detection and classification algorithms to uncover new signals and trends in ambient soundscapes.

The classifier was found to be robust against a manually annotated *Dascyllus albisella* dataset (AUC = 0.9), demonstrating the applicability of this model to other survey sites. While this model was specifically tailored to Hawaiian reefs, it can be readily retrained on other acoustic datasets in order to extract commonly occurring signals in those environments. The duration and frequency range of spectrogram samples were chosen to reflect the general characteristics of fish vocalizations in reefs (0.36 s, 150–750 Hz), however the model also detected and classified signals with durations and frequencies larger than the window ranges provided. Parrotfish scrapes have peak frequencies on the order of 2–4 kHz, but are broadband (200–8000 Hz) and commonly occupy the lower frequencies studied here [55,56]. Humpback song units typically have peak frequencies between 0-1.5 kHz, with song unit durations on the order of 1–3 s [e.g., 49,57]. Tonals from passing ships can last minutes or hours, depending on the source level, speed and distance of the ship [e.g., 48,58]. The model architecture can therefore be applied with minimal prior knowledge of the signals that comprise the soundscape and can be used to discover signals that were unknown to the human analyst.

It is important to note that the optimization of the detector and spectrogram parameters for fish vocalizations likely resulted in suboptimal detection performance for signals with different spectral and temporal characteristics. This tradeoff is evident for longer-duration tonal signals, where many of the “ship” detections were mislabeled seismic activity, and one of the ambiguous clusters (“cluster 18”) was found to contain a mixture of ship sounds and humpback sounds (S3 and S10 Figs). In addition, a large number of uncharacterized clusters contain short-duration (<0.02 s) pulses, which were comprised of a mixture of biological and non-biological transient sounds (S3 Fig).

Addressing these limitations presents clear opportunities for future refinement. For instance, the characterization of signals with varying bandwidths and durations could be improved by implementing parallel models with spectrogram parameters tailored to different signal types. Future work could also expand the quantitative testing of classification performance beyond our initial use of labeled damselfish sounds by creating new datasets to calibrate class-specific likelihood thresholds for other sound types. Classification accuracy could be further optimized using “human in the loop” approaches [1], where automatically generated clusters are manually refined or subdivided for supervised training. In this process, growing public libraries of identified fish sounds, such as FishSounds [59], the Global Library of Underwater Biological Sounds (GLUBS) [60], and the Macaulay Library (https://www.macaulaylibrary.org) would serve as valuable reference datasets for annotating and validating these clusters. Further, while *D. albisella* sounds are well-characterized and readily identifiable [42,43], the ability to extract identified sounds from a combined audio-video sensor array [2] highlights the opportunity to identify sound sources from the field, and then extract a large number of those species-labeled sounds for rapid retraining of machine learning models.

These results demonstrate the significant advantages offered by unsupervised machine learning for soundscape characterization and signal classification at scale. Acoustic signals in a long-term dataset can be ingested, parsed, and automatically clustered without the burden and expense of manual labeling. By successfully scaling an established autoencoder-GMM framework, this study demonstrates its power to extract meaningful, event-level ecological insights from complex, unlabeled acoustic data. In contrast to traditional supervised learning approaches, the detection and clustering architecture introduced here can be easily applied to a variety of unlabeled acoustic datasets. These methods can be particularly useful for datasets where individual sounds are complex or difficult to disentangle, or where the specific signals that comprise the underwater soundscape have not yet been characterized. Such computational efforts will be essential for providing actionable, taxonomically-specific sound identification to enable passive acoustic monitoring for management and conservation of coral reef fishes and ecosystems at global scales [6,61,62].

## Supporting information

S1 AudioAudio samples for each of the nine characterized classes.Audio files are produced for the nine characterized classes by collating ten randomly-selected one-second sound samples with a very high-likelihood score (L>0.999) for that class.(ZIP)

S1 Fig Non-convergence of traditional metrics for determining cluster count necessitates a modified AIC approach. Traditional clustering validation metrics including the Akaike Information Criterion (AIC), Bayesian Information Criterion (BIC), Silhouette score, and Calinski–Harabasz index were evaluated from cluster counts ranging from 10 to 45. To reduce computational costs, a random 10% subsample of the dataset was analyzed for clustering validation. The AIC, BIC, and Calinski–Harabasz scores trended downward with increasing cluster count, despite minor local fluctuations, while the Silhouette Score fluctuated from -0.07 to 0.01 across the surveyed range. The failure of these traditional metrics to converge on a single cluster count reflects the high proportion of noisy or ambiguous samples prevalent in complex coral reef soundscapes. Because these traditional indices struggle to resolve structure in highly noisy datasets, model selection required a targeted approach focused on data well-modeled by the Gaussian mixture model. We implemented a modified AIC, computed exclusively using samples within “well-defined” clusters (defined as clusters where >10% of assigned samples exhibited assignment likelihoods >0.99).
(PDF)

S2 FigRecreations of Fig 4, showing the effect of lowering the likelihood threshold for sample inclusion.For a sample to be included in the visualization for a given class, it must belong to that cluster and exceed a likelihood threshold L. The thresholds chosen are L>0.9 (A), L>0.5 (B), L>0.25 (C), L>0 (D). While consistent spectral and temporal features are preserved as the detection threshold is lowered, this adjustment leads to the inclusion of lower-SNR samples and an elevated risk of false positives.(PDF)

S3 FigRandomly selected spectrogram samples (L>0.9) for the 20 uncharacterized clusters, which lacked consistent, distinct biological or anthropogenic signatures.Many of these uncharacterized clusters (e.g., 6, 7, 8, 9, 12, 17, 20) contain short-duration (<0.02 s) pulses, which included a mixture of biological pulses and non-biological transient sounds. Other clusters (e.g., 1, 4, 5, 19) contained longer duration (>0.1 s) sounds which were again comprised of a diverse mixture of biological and non-biological transients. Clusters 2 and 13 contained low-SNR signals with few visible features in the spectrograms. Cluster 18 was found to contain a mixture of humpback whale sounds and ship tonals, which is confirmed by the diel/seasonal analysis shown in S10 Fig. A detection threshold of 0.9 was chosen since many of these uncharacterized clusters did not have samples with likelihoods above 0.99.(PDF)

S4 FigThe logarithm of the generalized variance for each of the 29 clusters automatically generated by the Gaussian mixture model, where the generalized variance is calculated as the determinant of the covariance matrix Σ.The 29 clusters show log|Σ| values ranging from approximately 30–60. The damselfish2 and holocentrid classes have the greatest generalized variance values (log|Σ| > 55), suggesting that these sound classes contain a diverse range of vocalizations. The lowest generalized variance values (log|Σ| < 35) are associated with the parrotfish class and clusters 13, 17, and 18, suggesting a narrower range of sound types.(PNG)

S5 Fig Matrix showing the Bhattacharyya coefficient for each cluster pair within the 29 automatically generated clusters. The Bhattacharyya coefficient is a measure of the similarity between probability distributions, ranging from 0 to 1. The highest coefficient of 0.50 occurs between the unknown fish class and cluster 14, suggesting moderate overlap. However, as shown in S6 Fig, cluster 14 has a substantially lower proportion of high-likelihood (>0.99) samples compared to the unknown fish class (<0.001% vs. > 10%, respectively), which indicates that the inclusion of cluster 14 would not significantly impact classification performance for high-confidence detections. The Bhattacharyya coefficients for every other cluster pair are less than 0.36, indicating minimal overlap between probability distributions.
(PNG)

S6 Fig Distribution of likelihood scores for all samples within each of the 29 automatically generated clusters. The nine characterized clusters have prominent modes above 0.99, indicating a high percentage of high-confidence detections. In contrast, many of the uncharacterized clusters (numbered 1–20) lack a high-confidence peak, with distributions centered around a likelihood of 0.5. This suggests that these clusters may contain a high proportion of ambiguous or noisy samples with uncertain assignments. However, a subset of uncharacterized clusters (e.g., 1, 2, 9, 19, 20) also exhibit high-confidence peaks, suggesting they represent acoustically consistent signals.
(PNG)

S7 FigComparison of detection performance between k-means and Gaussian mixture modeling.We benchmarked the detection performance of Gaussian mixture modeling (GMM) against k-means clustering, using the labeled damselfish calls as ground truth. Latent representations from the training dataset (7,767,943 samples) were partitioned using k-means with 29 clusters to match the number of GMM clusters and ensure a mathematically fair comparison. Latent representations from the labeled test dataset (6,098 samples) were then clustered using the pre-trained k-means algorithm, with no extra training on the labeled dataset. The k-means clusters were ranked in descending order based on the number of labeled damselfish calls they contained, and test samples were classified as damselfish if they mapped to the top n clusters, where n serves as an integer detection threshold. ROC curves and Precision-Recall curves were generated for the k-means clusters by varying n from 0 to 29. GMM outperformed k-means across both evaluation frameworks, achieving an ROC Area Under the Curve (AUC) of 0.9 compared to 0.85 for k-means (a) and a Precision-Recall AUC of 0.32 compared to 0.23 for k-means (b).(PNG)

S8 FigRecreations of Fig 7, showing the effect of lowering the likelihood threshold for sample inclusion.For a sample to be included in the visualization for a given class, it must belong to that cluster and exceed a likelihood threshold L. The thresholds shown are L>0.9 (A), L>0.5 (B), L>0.25 (C), L>0 (D). The diel, lunar, and seasonal trends seen in Fig 7 are mostly preserved as the threshold is lowered. The primary exception is the “ship” class, where a significantly lowered threshold results in elevated nighttime detections, including sunset activity likely associated with biological sound (possible confusion with the “unknown fish” sound).(PDF)

S9 Fig Diel and seasonal trends for the nine characterized classes at all survey sites. Detection rates are shown as a function of time of year (vertical axis) and time of day (horizontal axis). Solid yellow and cyan lines respectively correspond to sunrise and sunset.
(PDF)

S10 Fig Diel and seasonal trends for cluster 18, which is found to correspond to a mixture of humpback whale vocalizations and ship sounds. In the survey site where humpback vocalizations are most frequent (Site 1), detections primarily occur between the months of December and April, consistent with the seasonal pattern seen for classes “humpback1,2,3.” In the site where ship sounds are most frequent (Site 4), detections occurred at sporadic intervals mostly during daytime hours, consistent with the diel pattern seen for the “ship” class. Detections in Sites 2 and 3 contained a mixture of the seasonal humpback pattern and the diel ship pattern.
(PNG)
