# Supplementary figures and images for: Multi-class, unsupervised detection and classification of biological and anthropogenic sounds in coral reefs
Source: PLoS Comput Biol. 2026 Jul 20;22(7):e1014516. doi: 10.1371/journal.pcbi.1014516 (PMC13411937; doi:10.1371/journal.pcbi.1014516)

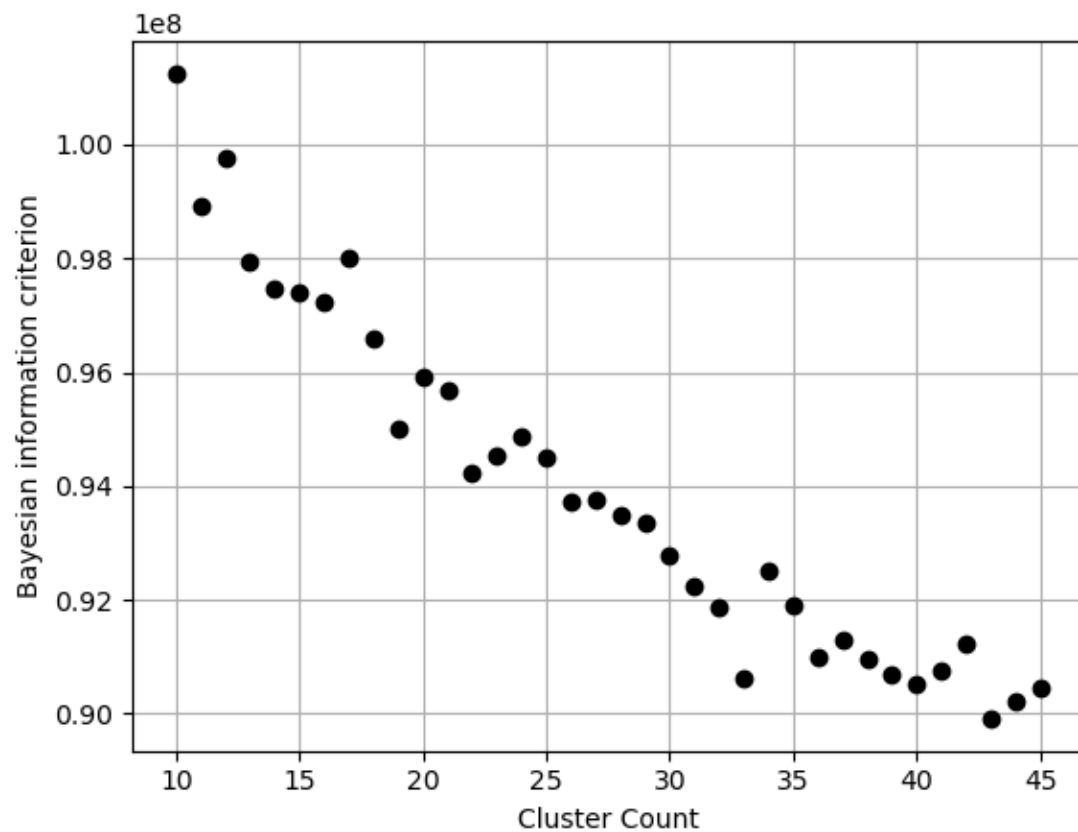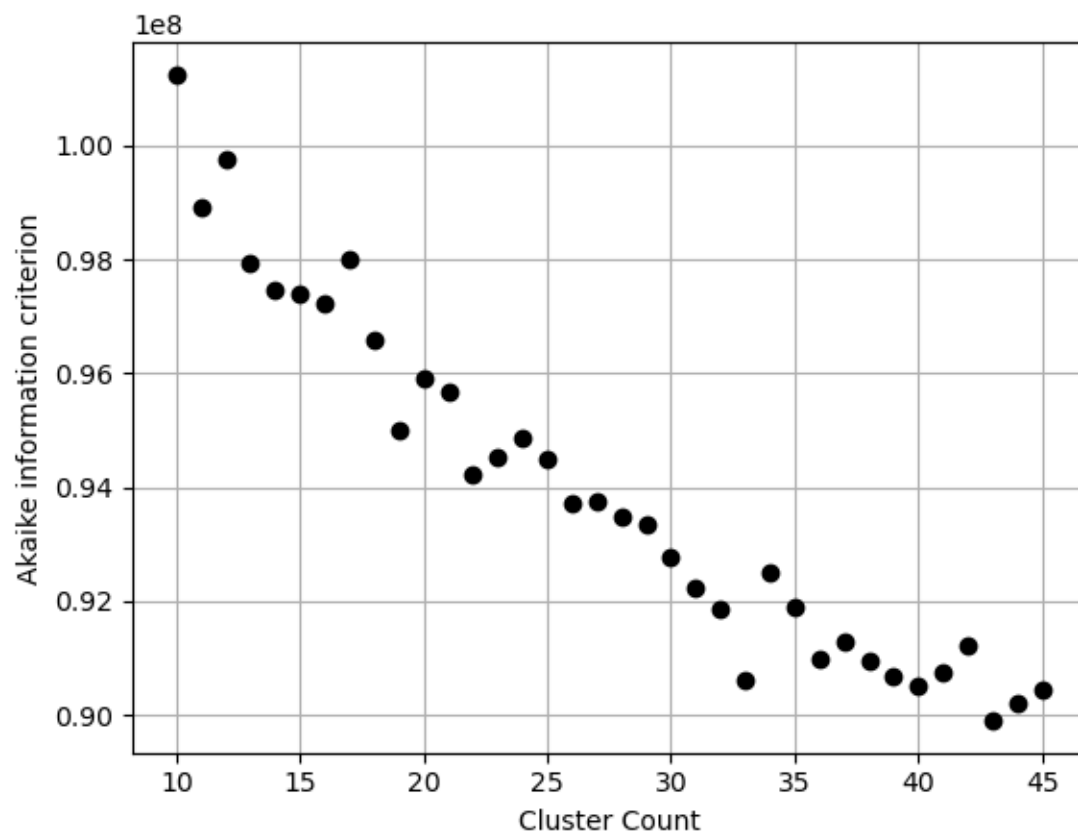

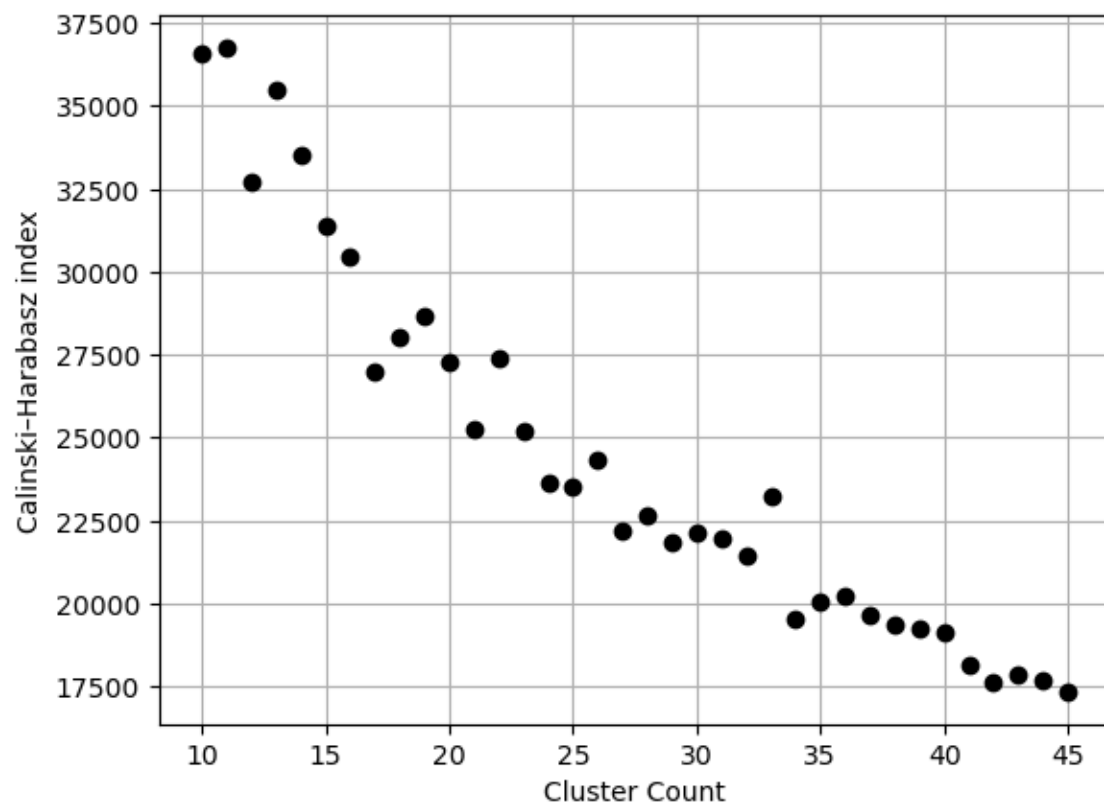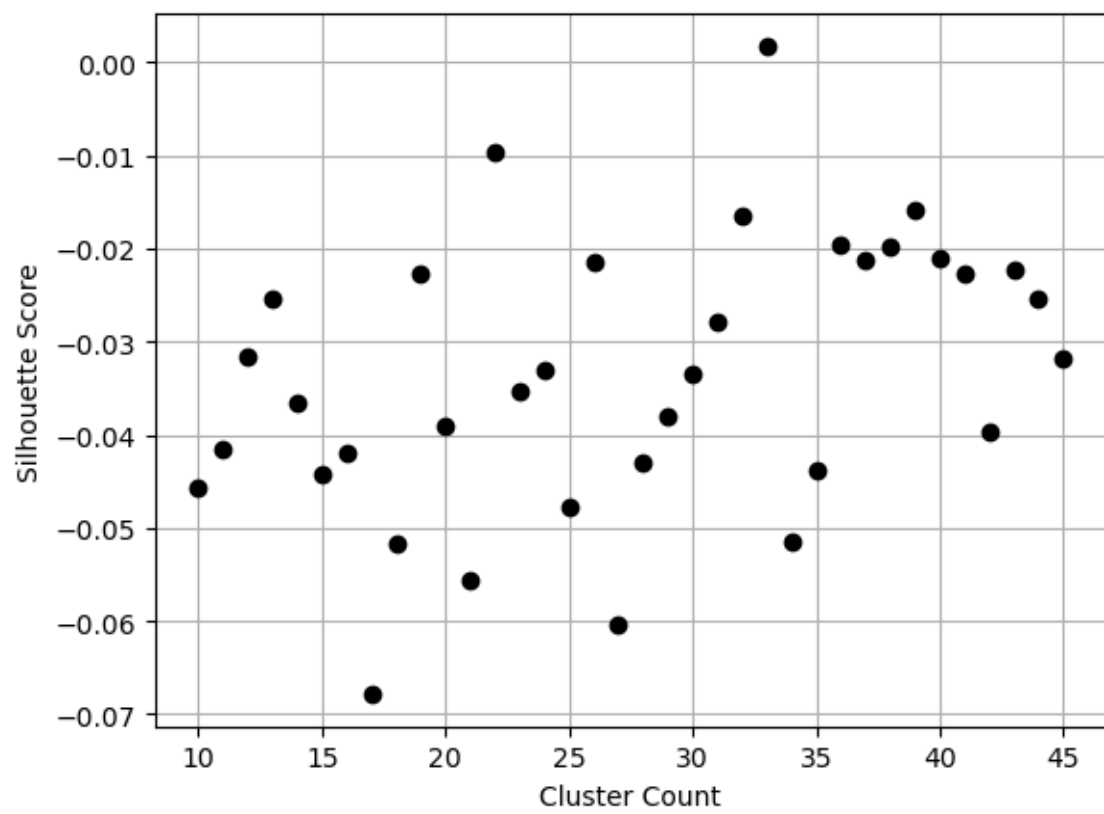

Supplement: S1 Fig — Traditional clustering validation metrics including the Akaike Information Criterion (AIC), Bayesian Information Criterion (BIC), Silhouette score, and Calinski–Harabasz index were evaluated from cluster counts ranging from 10 to 45. To reduce computational costs, a random 10% subsample of the dataset was analyzed for clustering validation. The AIC, BIC, and Calinski–Harabasz scores trended downward with increasing cluster count, despite minor local fluctuations, while the Silhouette Score fluctuated from -0.07 to 0.01 across the surveyed range. The failure of these traditional metrics to converge on a single cluster count reflects the high proportion of noisy or ambiguous samples prevalent in complex coral reef soundscapes. Because these traditional indices struggle to resolve structure in highly noisy datasets, model selection required a targeted approach focused on data well-modeled by the Gaussian mixture model. We implemented a modified AIC, computed exclusively using samples within “well-defined” clusters (defined as clusters where >10% of assigned samples exhibited assignment likelihoods >0.99). (PDF) [file pcbi.1014516.s002.pdf]

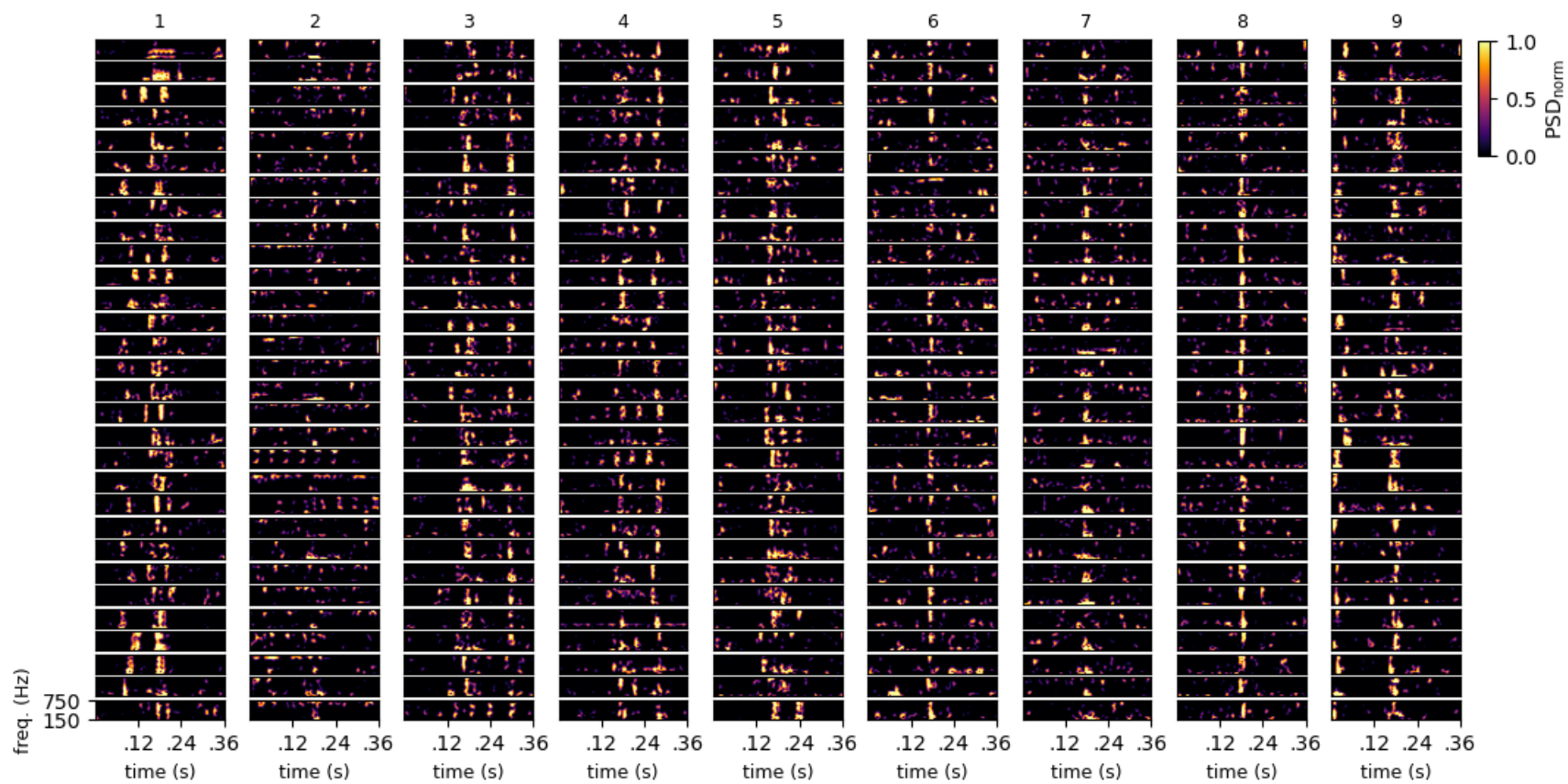

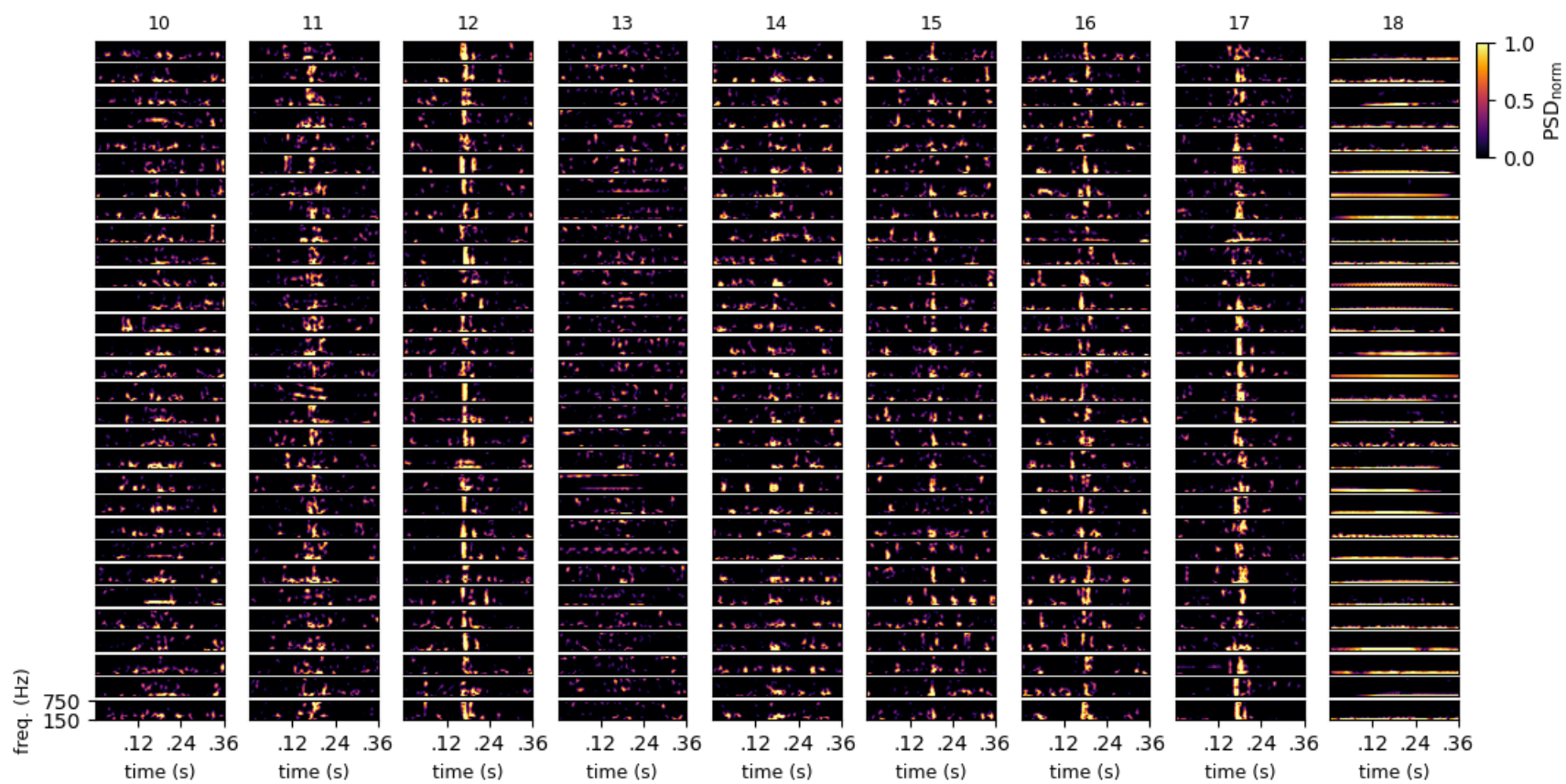

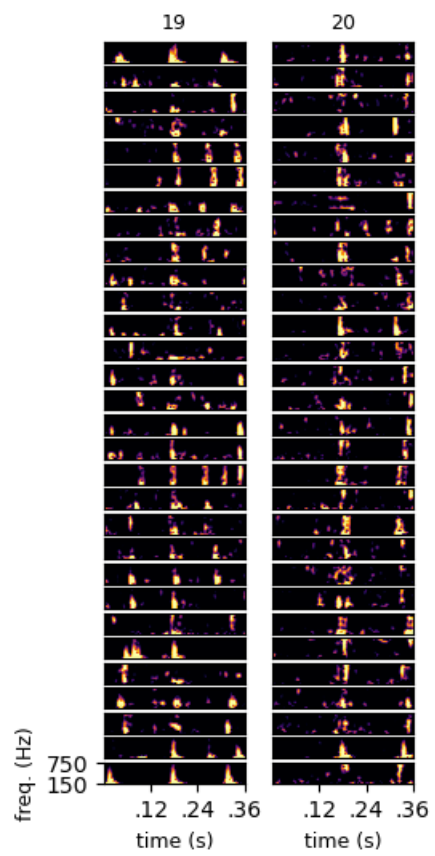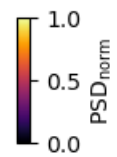

Supplement: S3 Fig — Many of these uncharacterized clusters (e.g., 6, 7, 8, 9, 12, 17, 20) contain short-duration (<0.02 s) pulses, which included a mixture of biological pulses and non-biological transient sounds. Other clusters (e.g., 1, 4, 5, 19) contained longer duration (>0.1 s) sounds which were again comprised of a diverse mixture of biological and non-biological transients. Clusters 2 and 13 contained low-SNR signals with few visible features in the spectrograms. Cluster 18 was found to contain a mixture of humpback whale sounds and ship tonals, which is confirmed by the diel/seasonal analysis shown in S10 Fig. A detection threshold of 0.9 was chosen since many of these uncharacterized clusters did not have samples with likelihoods above 0.99. (PDF) [file pcbi.1014516.s004.pdf]

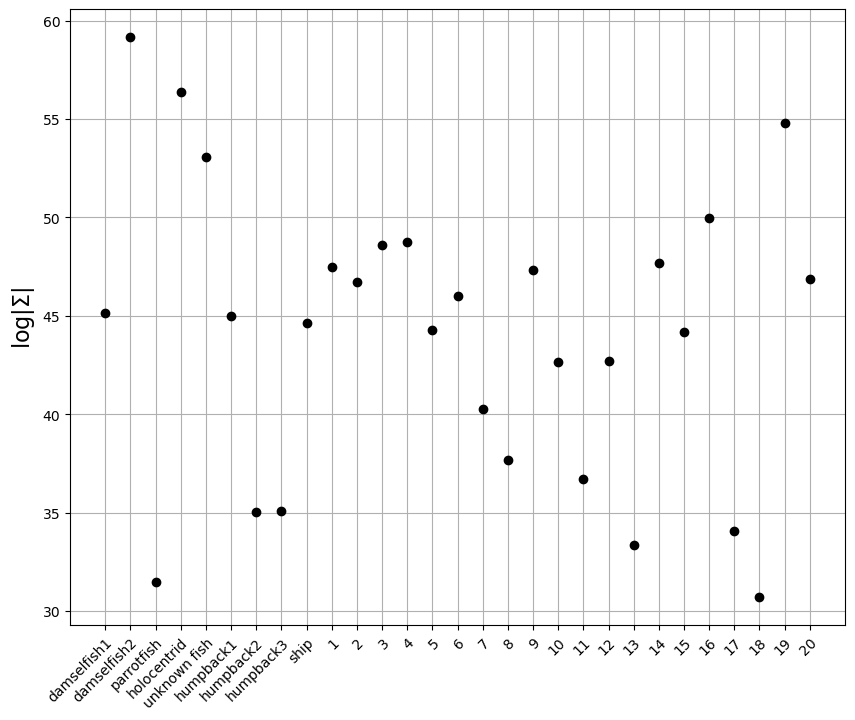

Supplement: S4 Fig — The 29 clusters show log|Σ| values ranging from approximately 30–60. The damselfish2 and holocentrid classes have the greatest generalized variance values (log|Σ| > 55), suggesting that these sound classes contain a diverse range of vocalizations. The lowest generalized variance values (log|Σ| < 35) are associated with the parrotfish class and clusters 13, 17, and 18, suggesting a narrower range of sound types. (PNG) [file pcbi.1014516.s005.png]

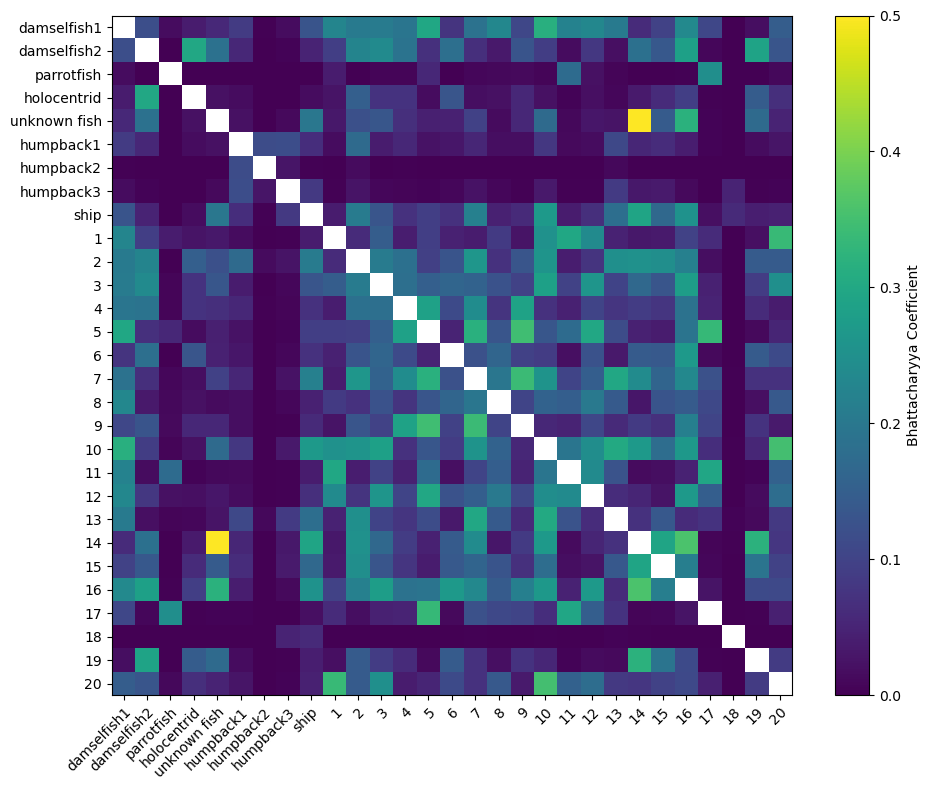

Supplement: S5 Fig — The Bhattacharyya coefficient is a measure of the similarity between probability distributions, ranging from 0 to 1. The highest coefficient of 0.50 occurs between the unknown fish class and cluster 14, suggesting moderate overlap. However, as shown in S6 Fig, cluster 14 has a substantially lower proportion of high-likelihood (>0.99) samples compared to the unknown fish class (<0.001% vs. > 10%, respectively), which indicates that the inclusion of cluster 14 would not significantly impact classification performance for high-confidence detections. The Bhattacharyya coefficients for every other cluster pair are less than 0.36, indicating minimal overlap between probability distributions. (PNG) [file pcbi.1014516.s006.png]

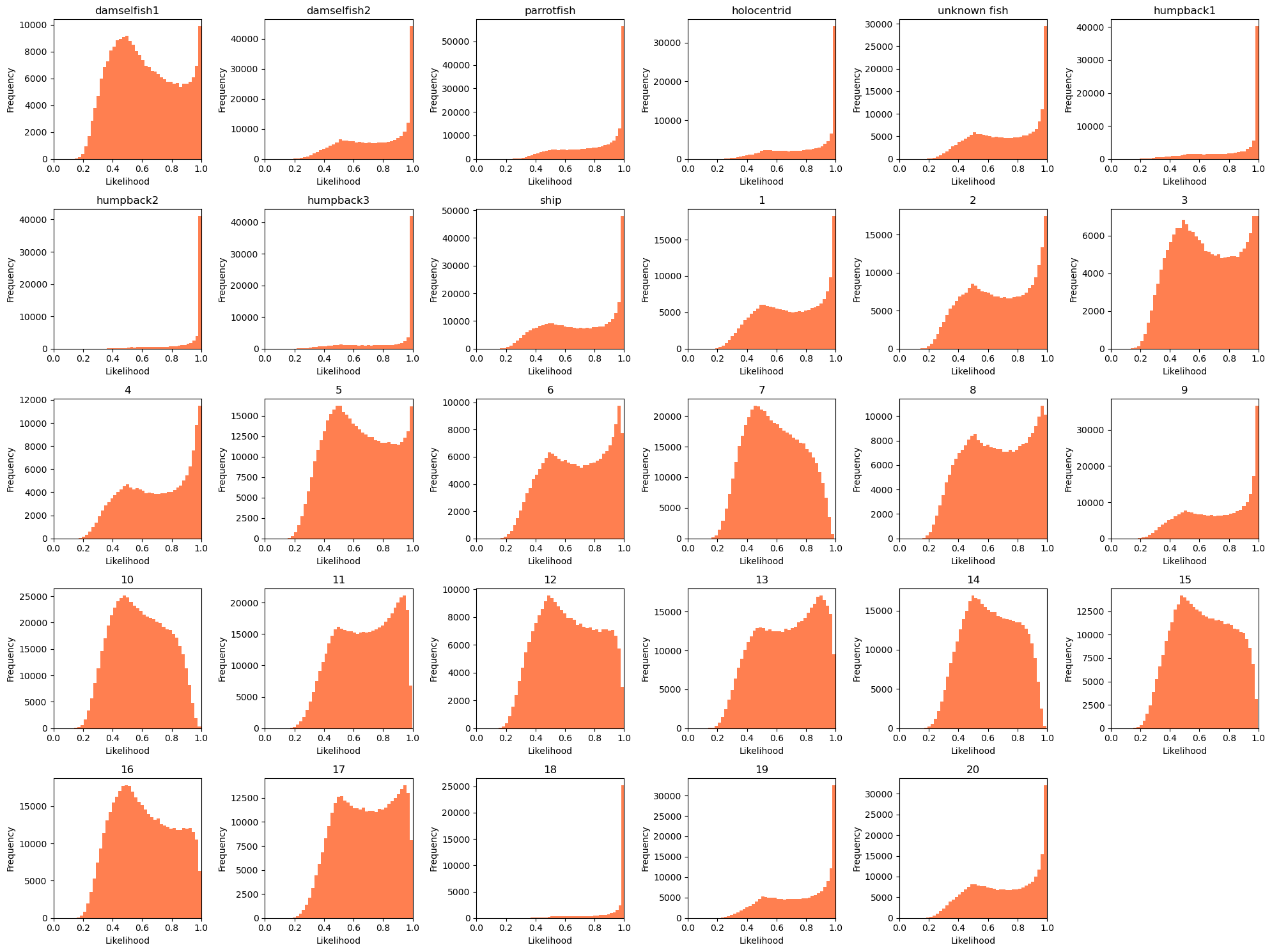

Supplement: S6 Fig — The nine characterized clusters have prominent modes above 0.99, indicating a high percentage of high-confidence detections. In contrast, many of the uncharacterized clusters (numbered 1–20) lack a high-confidence peak, with distributions centered around a likelihood of 0.5. This suggests that these clusters may contain a high proportion of ambiguous or noisy samples with uncertain assignments. However, a subset of uncharacterized clusters (e.g., 1, 2, 9, 19, 20) also exhibit high-confidence peaks, suggesting they represent acoustically consistent signals. (PNG) [file pcbi.1014516.s007.png]

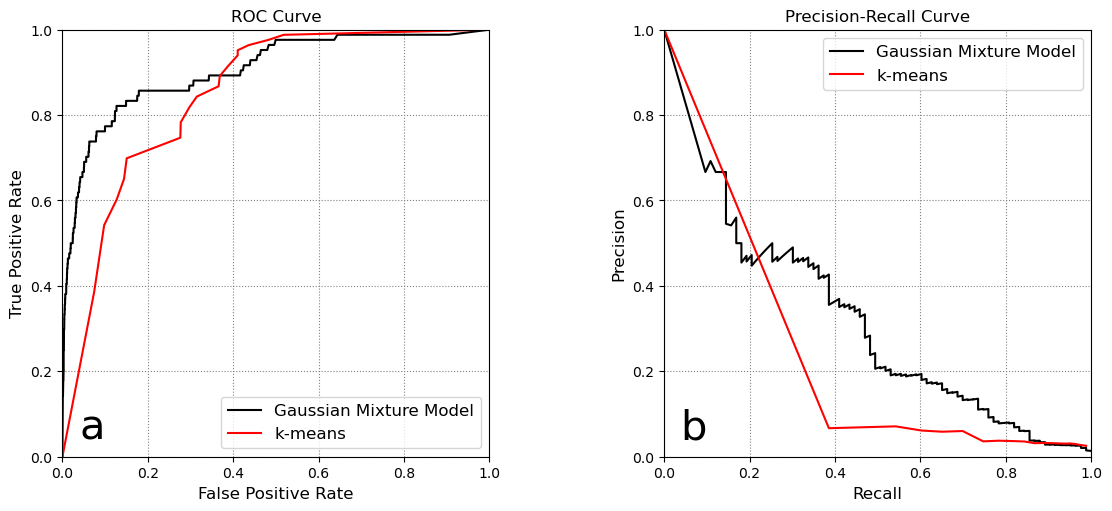

Supplement: S7 Fig — We benchmarked the detection performance of Gaussian mixture modeling (GMM) against k-means clustering, using the labeled damselfish calls as ground truth. Latent representations from the training dataset (7,767,943 samples) were partitioned using k-means with 29 clusters to match the number of GMM clusters and ensure a mathematically fair comparison. Latent representations from the labeled test dataset (6,098 samples) were then clustered using the pre-trained k-means algorithm, with no extra training on the labeled dataset. The k-means clusters were ranked in descending order based on the number of labeled damselfish calls they contained, and test samples were classified as damselfish if they mapped to the top n clusters, where n serves as an integer detection threshold. ROC curves and Precision-Recall curves were generated for the k-means clusters by varying n from 0 to 29. GMM outperformed k-means across both evaluation frameworks, achieving an ROC Area Under the Curve (AUC) of 0.9 compared to 0.85 for k-means (a) and a Precision-Recall AUC of 0.32 compared to 0.23 for k-means (b). (PNG) [file pcbi.1014516.s008.png]

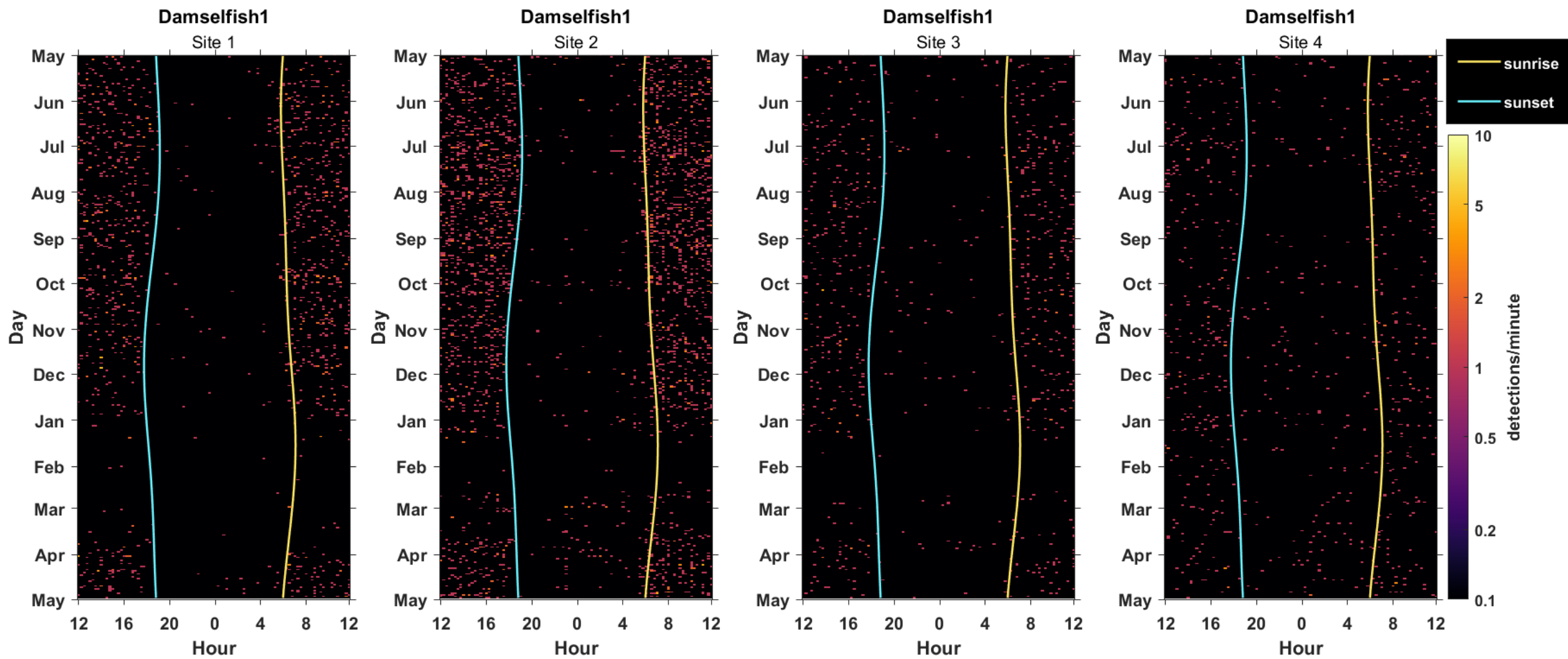

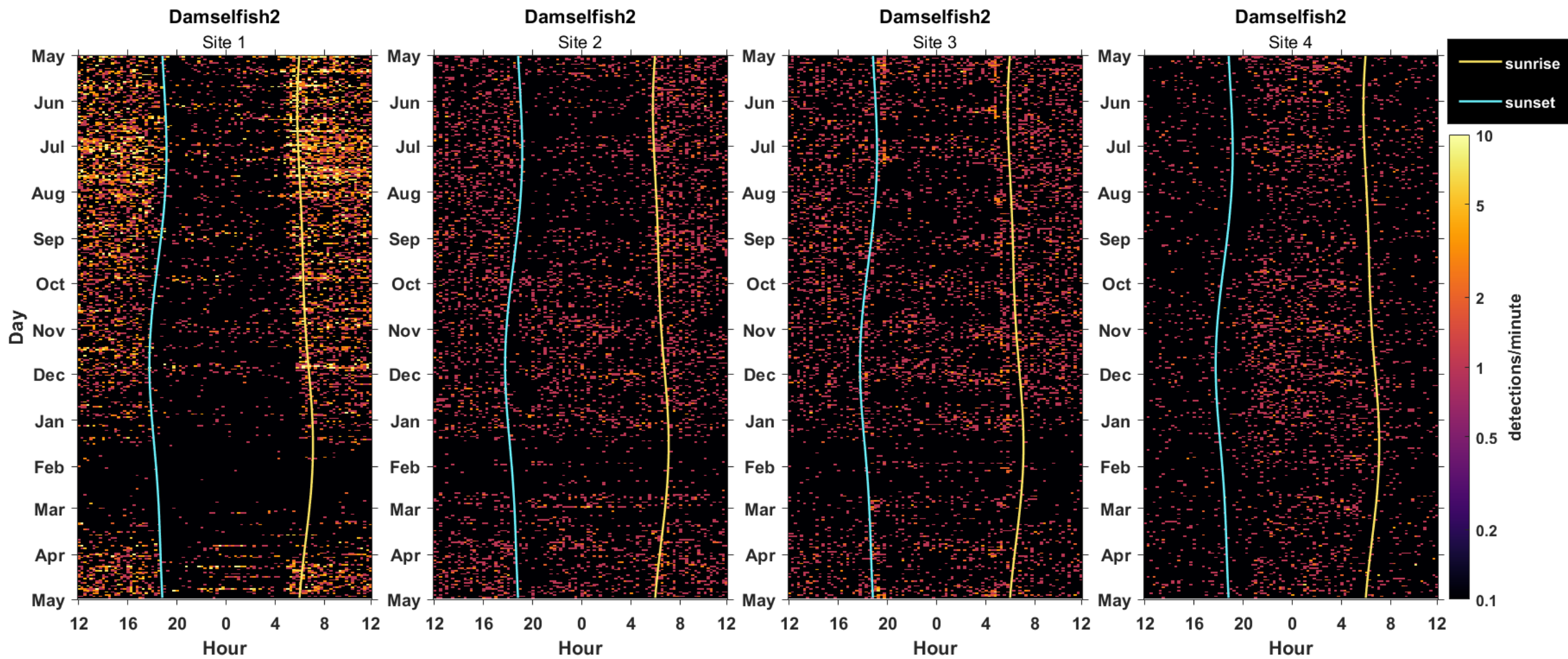

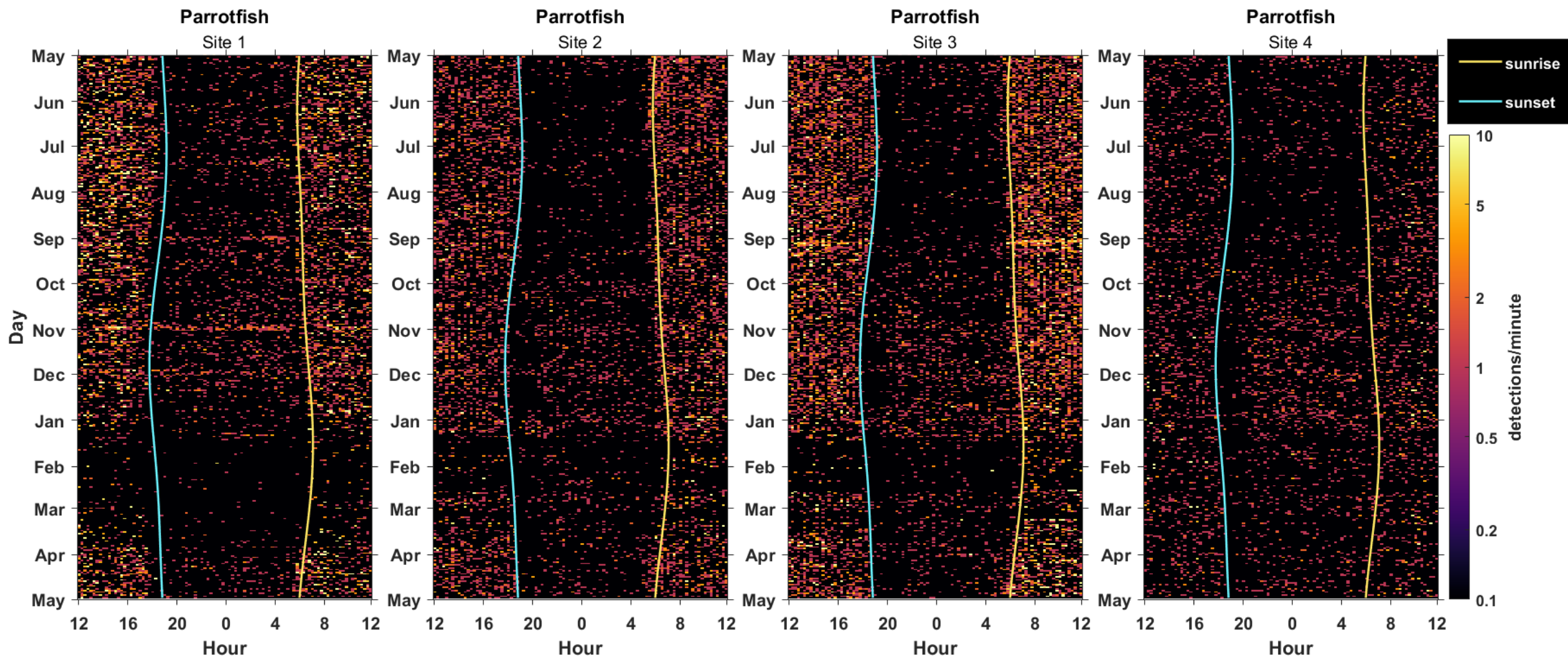

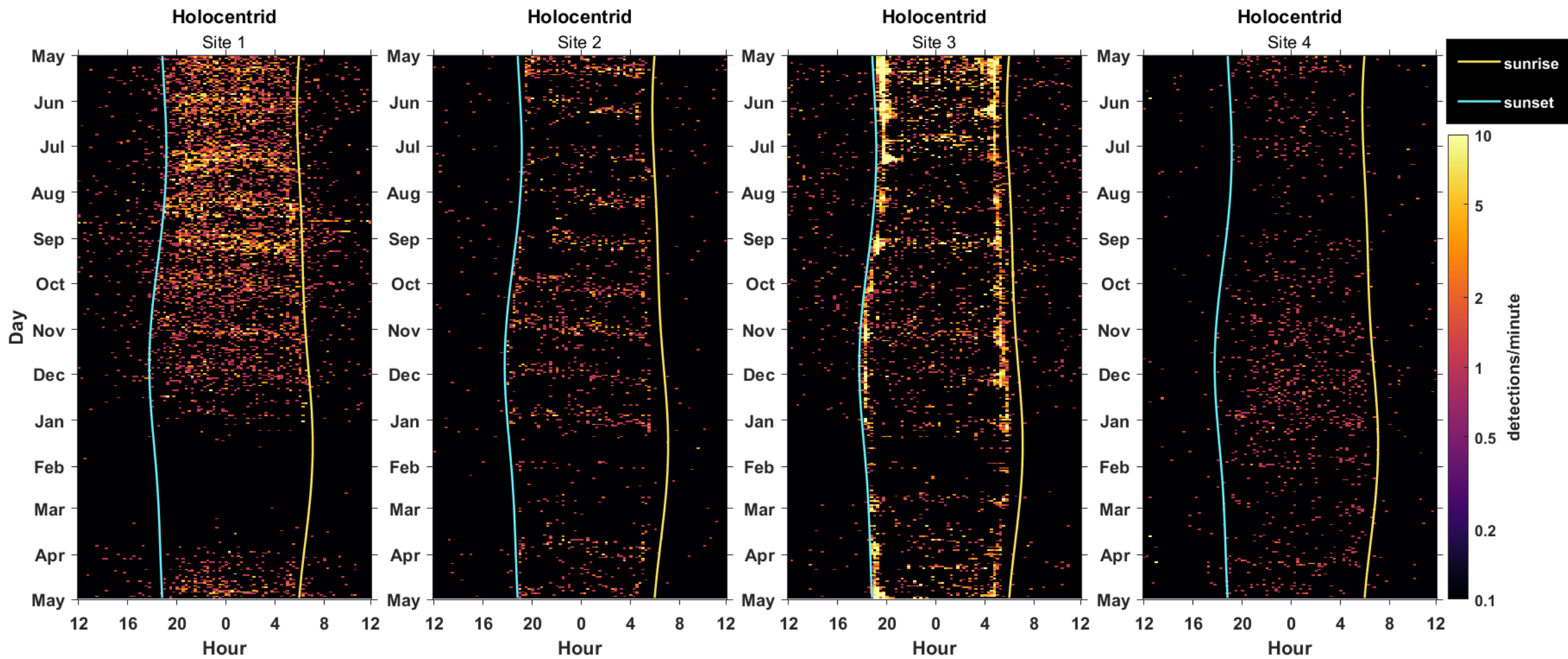

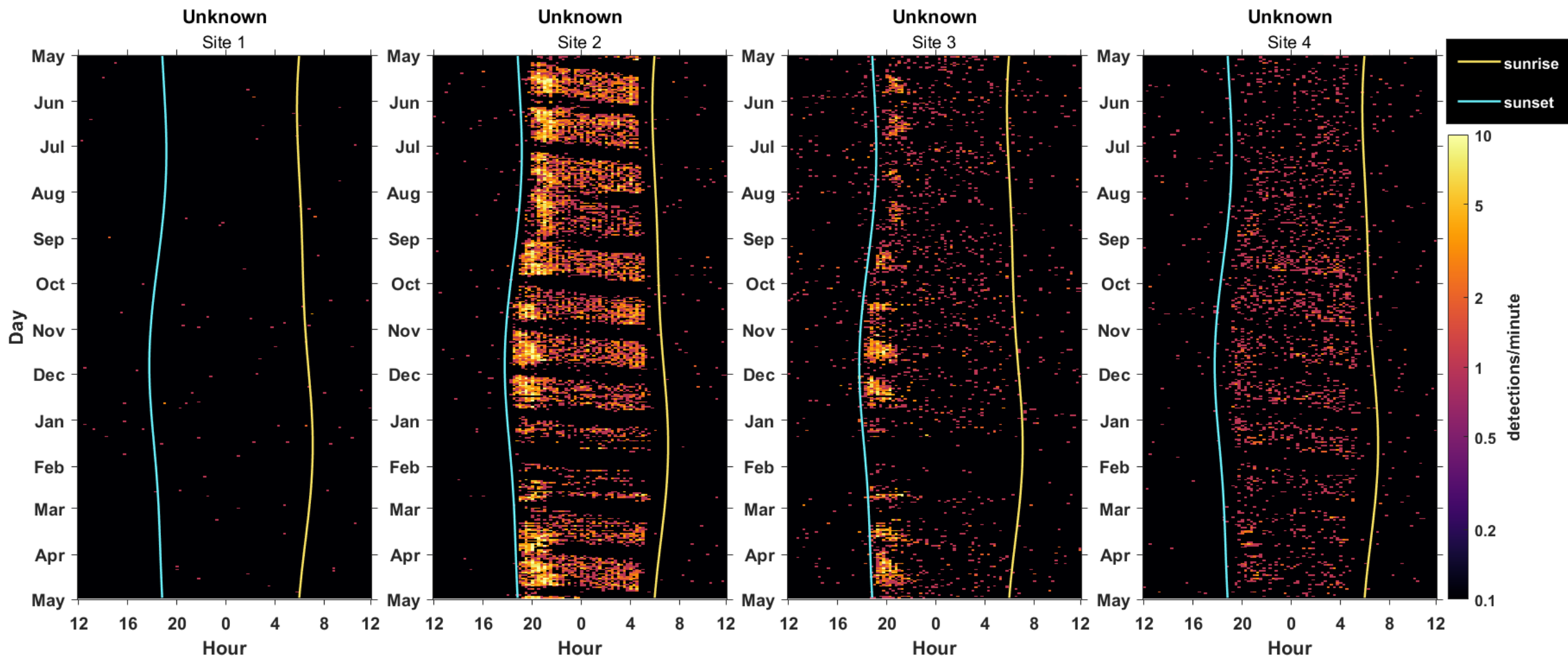

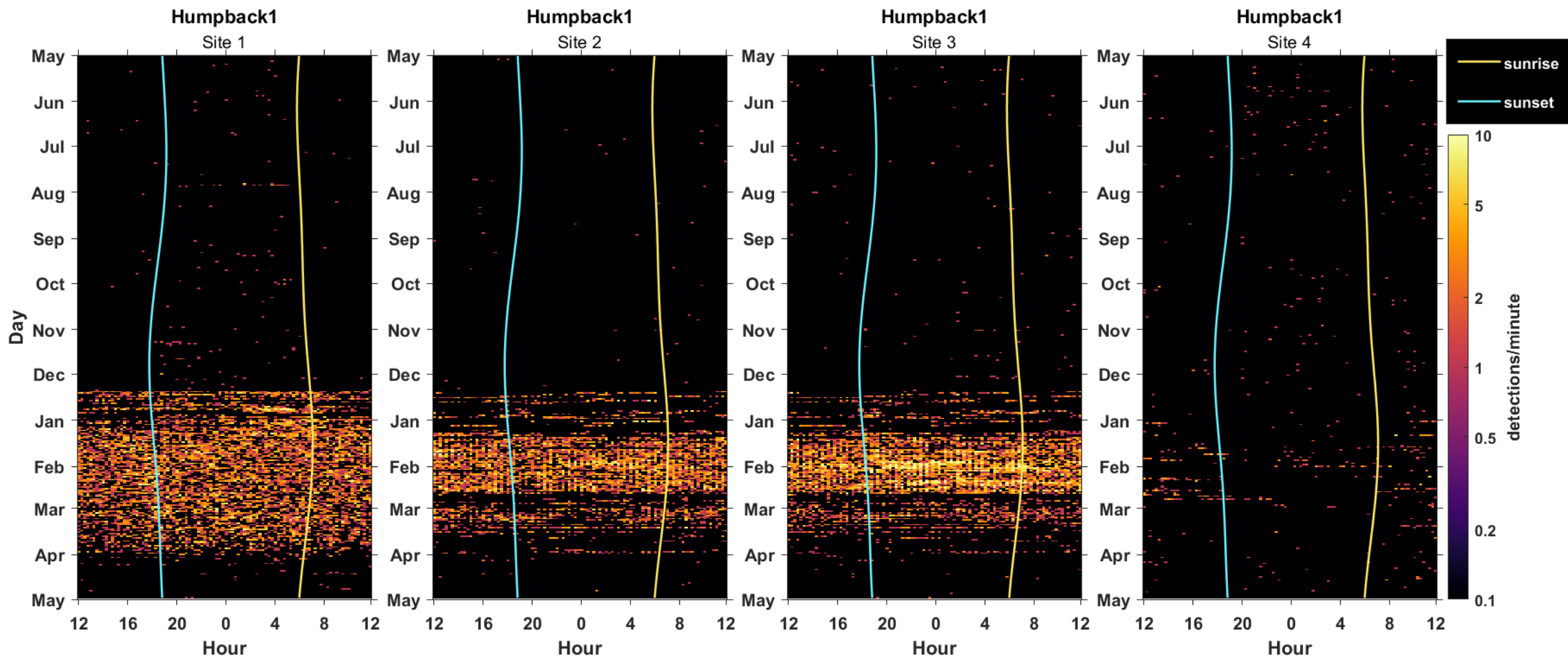

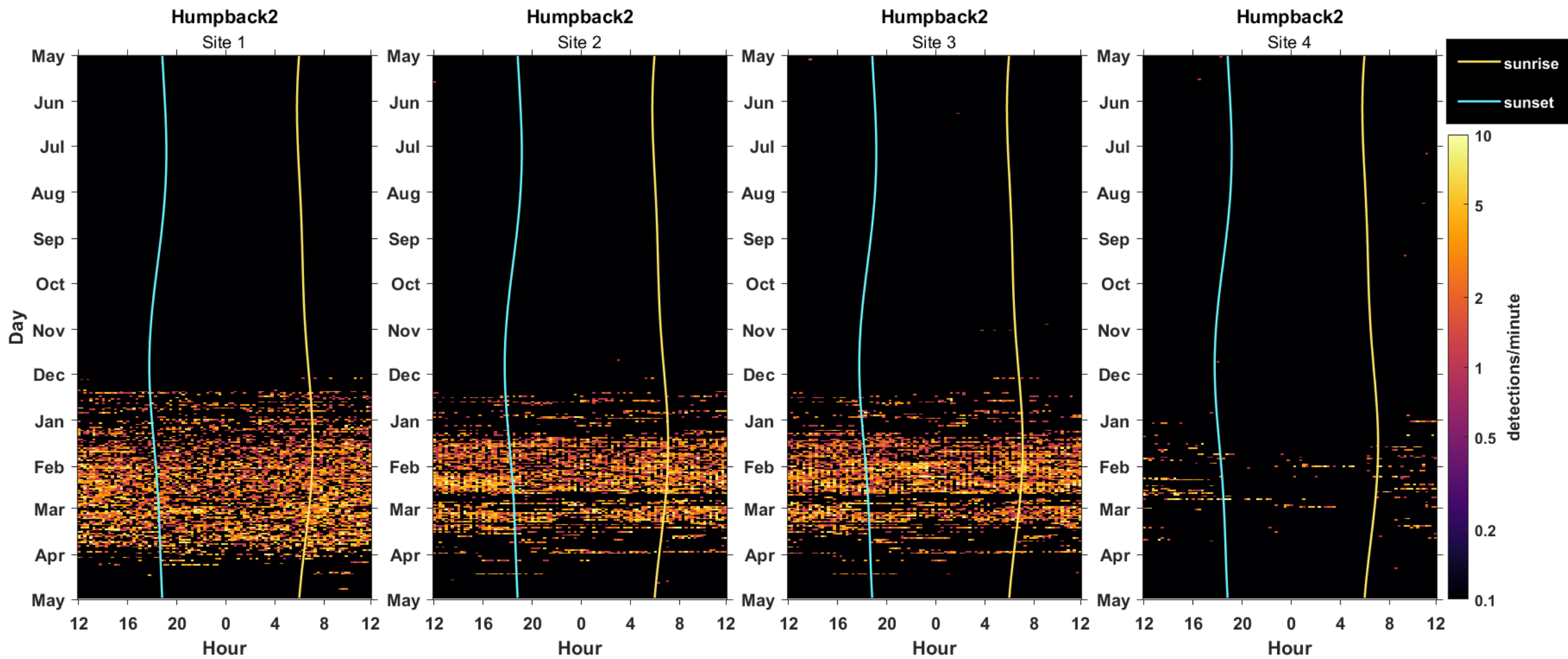

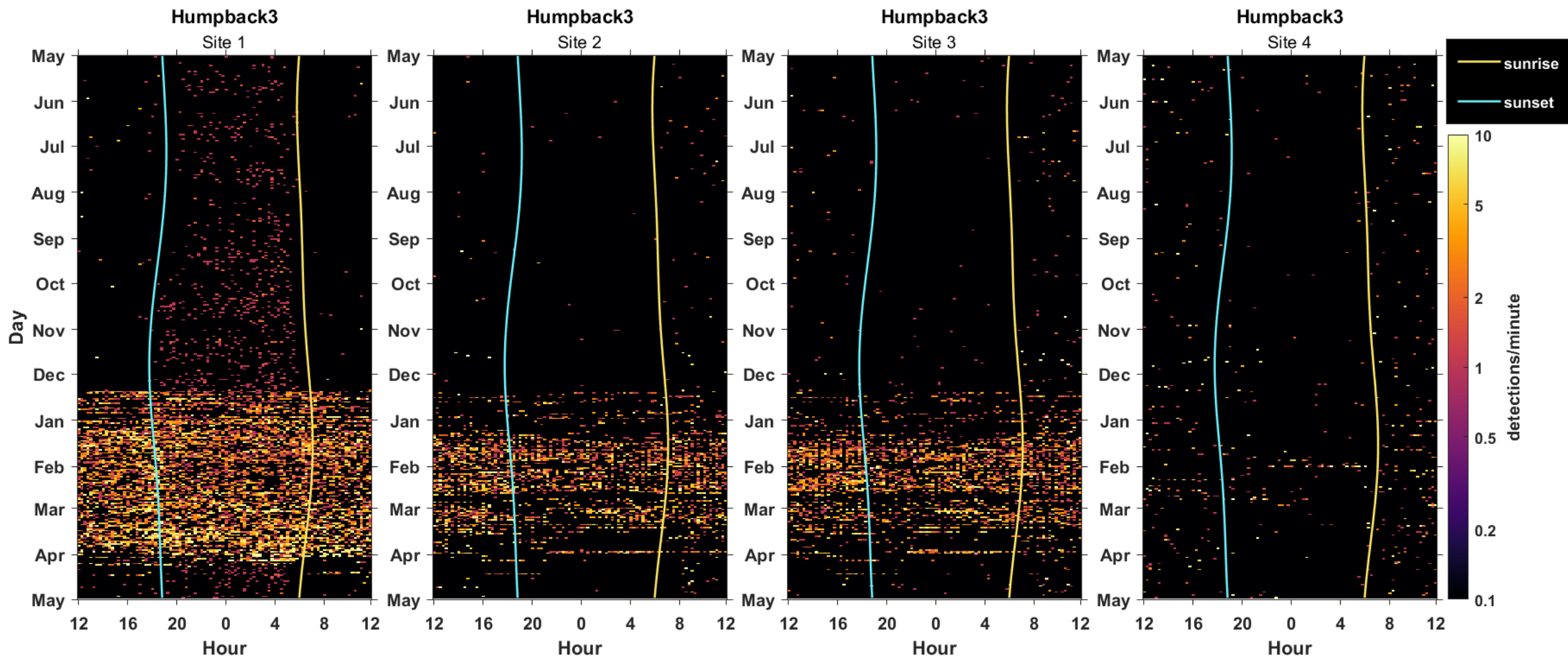

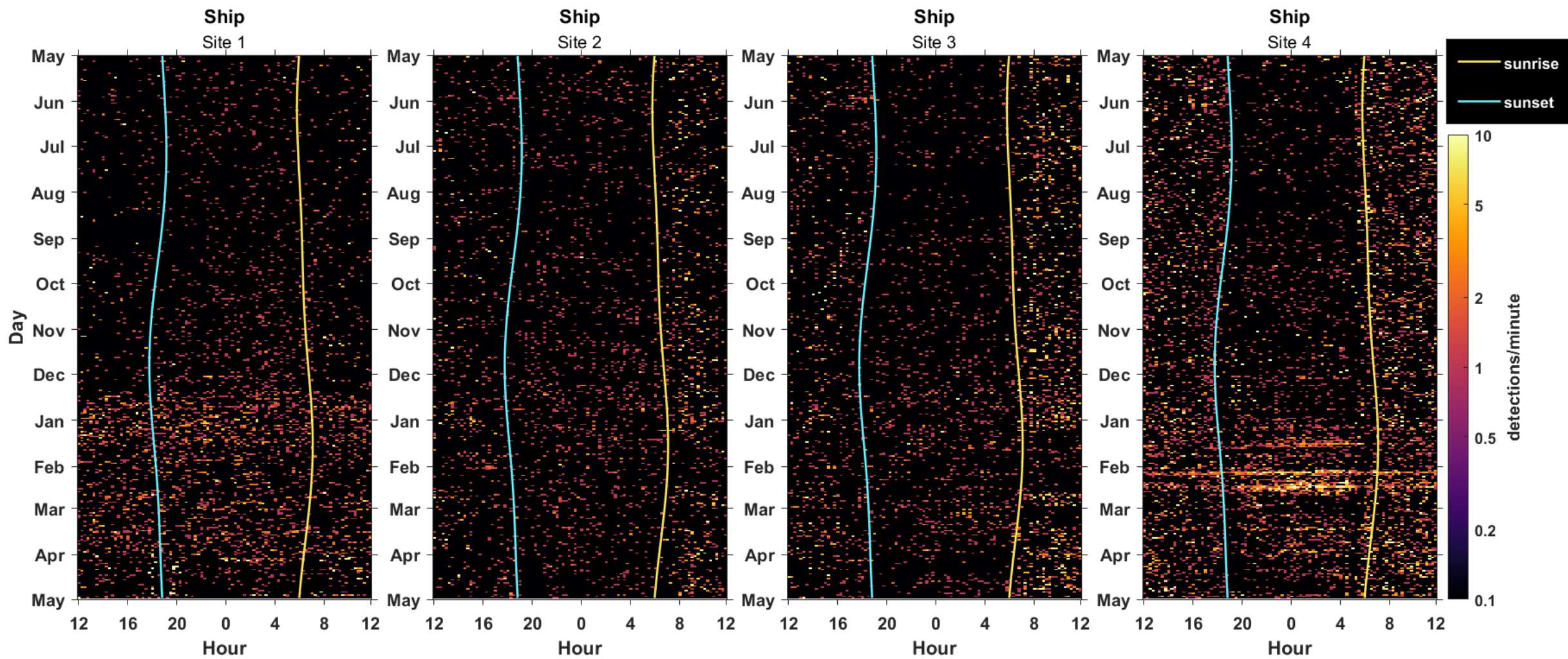

Supplement: S9 Fig — Detection rates are shown as a function of time of year (vertical axis) and time of day (horizontal axis). Solid yellow and cyan lines respectively correspond to sunrise and sunset. (PDF) [file pcbi.1014516.s010.pdf]

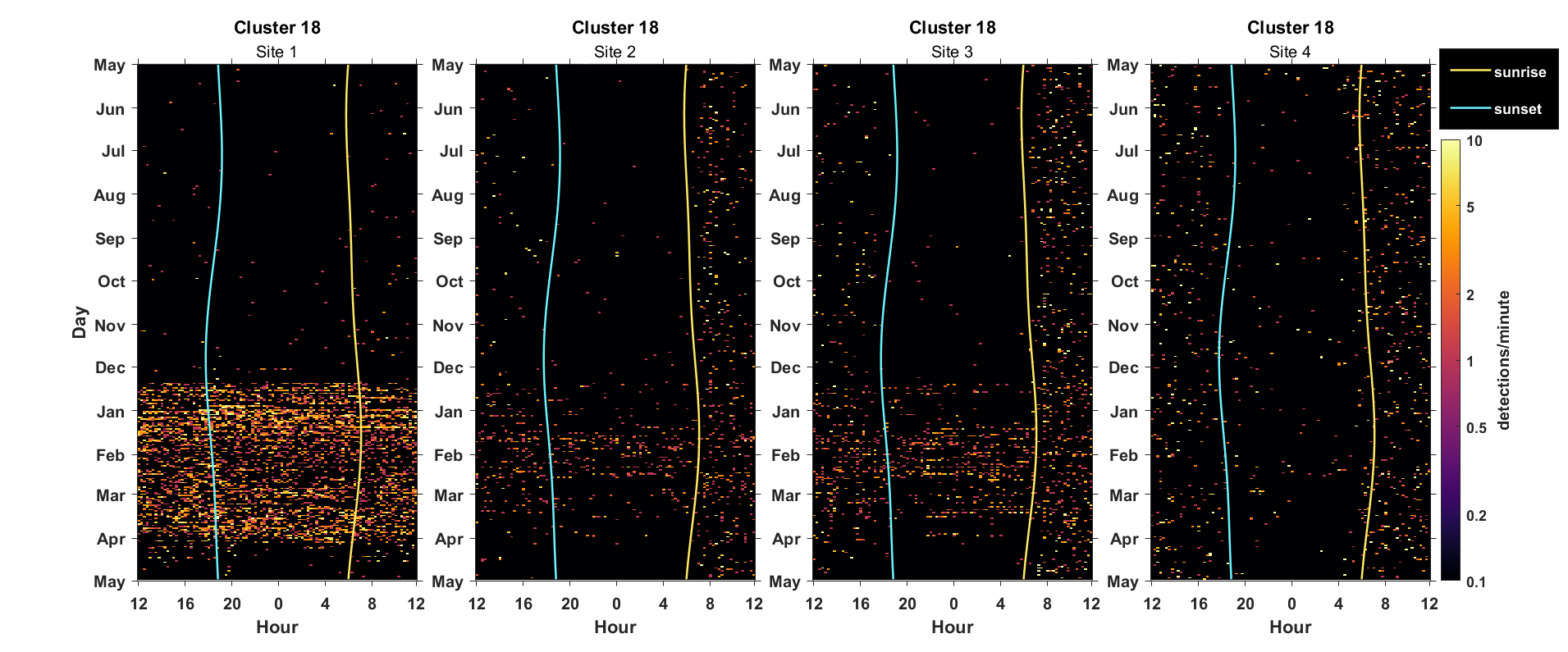

Supplement: S10 Fig — In the survey site where humpback vocalizations are most frequent (Site 1), detections primarily occur between the months of December and April, consistent with the seasonal pattern seen for classes “humpback1,2,3.” In the site where ship sounds are most frequent (Site 4), detections occurred at sporadic intervals mostly during daytime hours, consistent with the diel pattern seen for the “ship” class. Detections in Sites 2 and 3 contained a mixture of the seasonal humpback pattern and the diel ship pattern. (PNG) [file pcbi.1014516.s011.png]
