# Supplementary material for: Multi-class, unsupervised detection and classification of biological and anthropogenic sounds in coral reefs
Source: PLoS Comput Biol. 2026 Jul 20;22(7):e1014516. doi: 10.1371/journal.pcbi.1014516 (PMC13411937; doi:10.1371/journal.pcbi.1014516)
Supplement: S2 Fig — For a sample to be included in the visualization for a given class, it must belong to that cluster and exceed a likelihood threshold L. The thresholds chosen are L>0.9 (A), L>0.5 (B), L>0.25 (C), L>0 (D). While consistent spectral and temporal features are preserved as the detection threshold is lowered, this adjustment leads to the inclusion of lower-SNR samples and an elevated risk of false positives. (PDF) [file pcbi.1014516.s003.pdf]

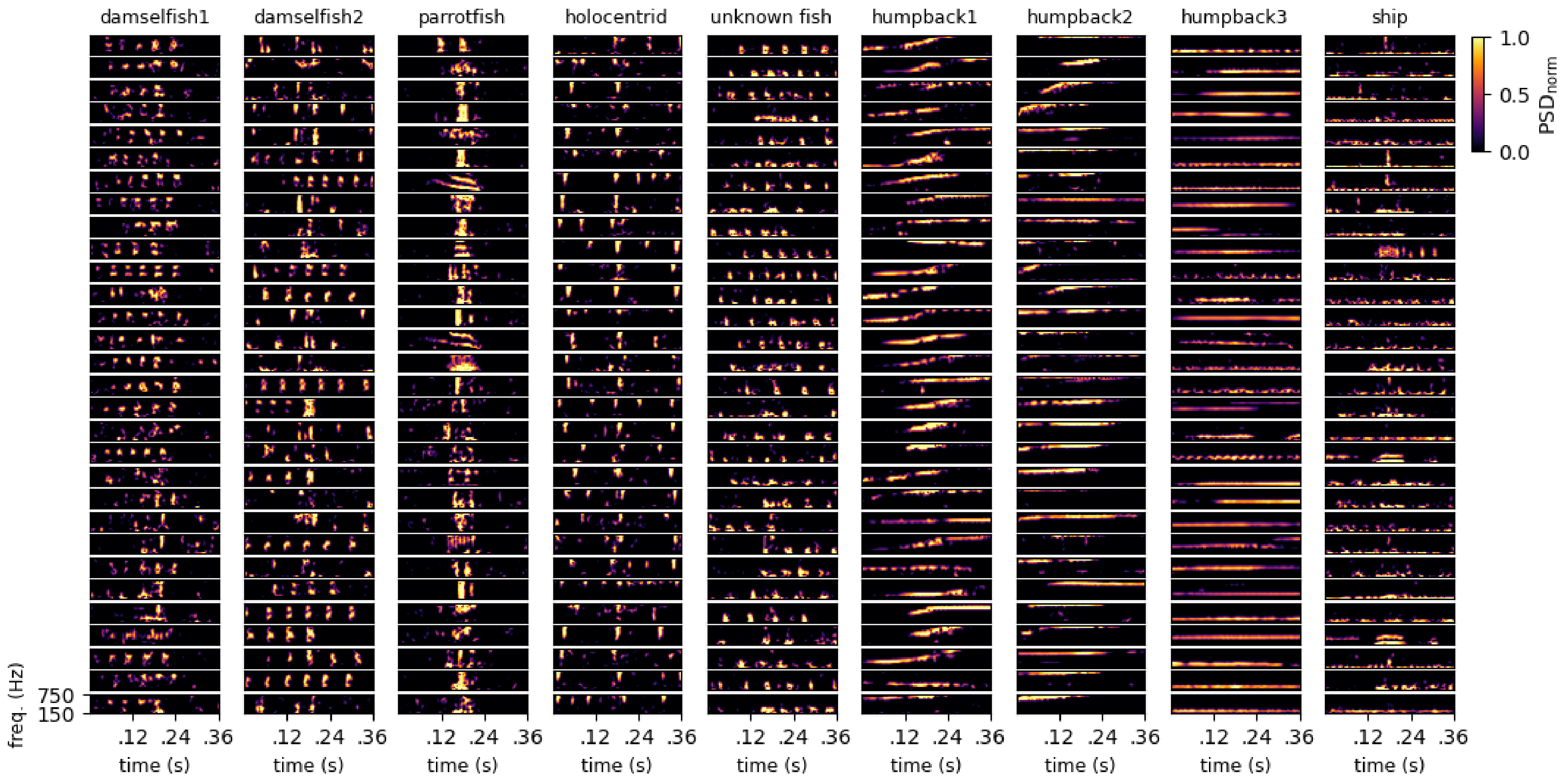

Figure S1A: Recreation of Figure 4 with spectrograms for randomly-selected sample detections in each cluster with likelihood scores greater than 0.9.

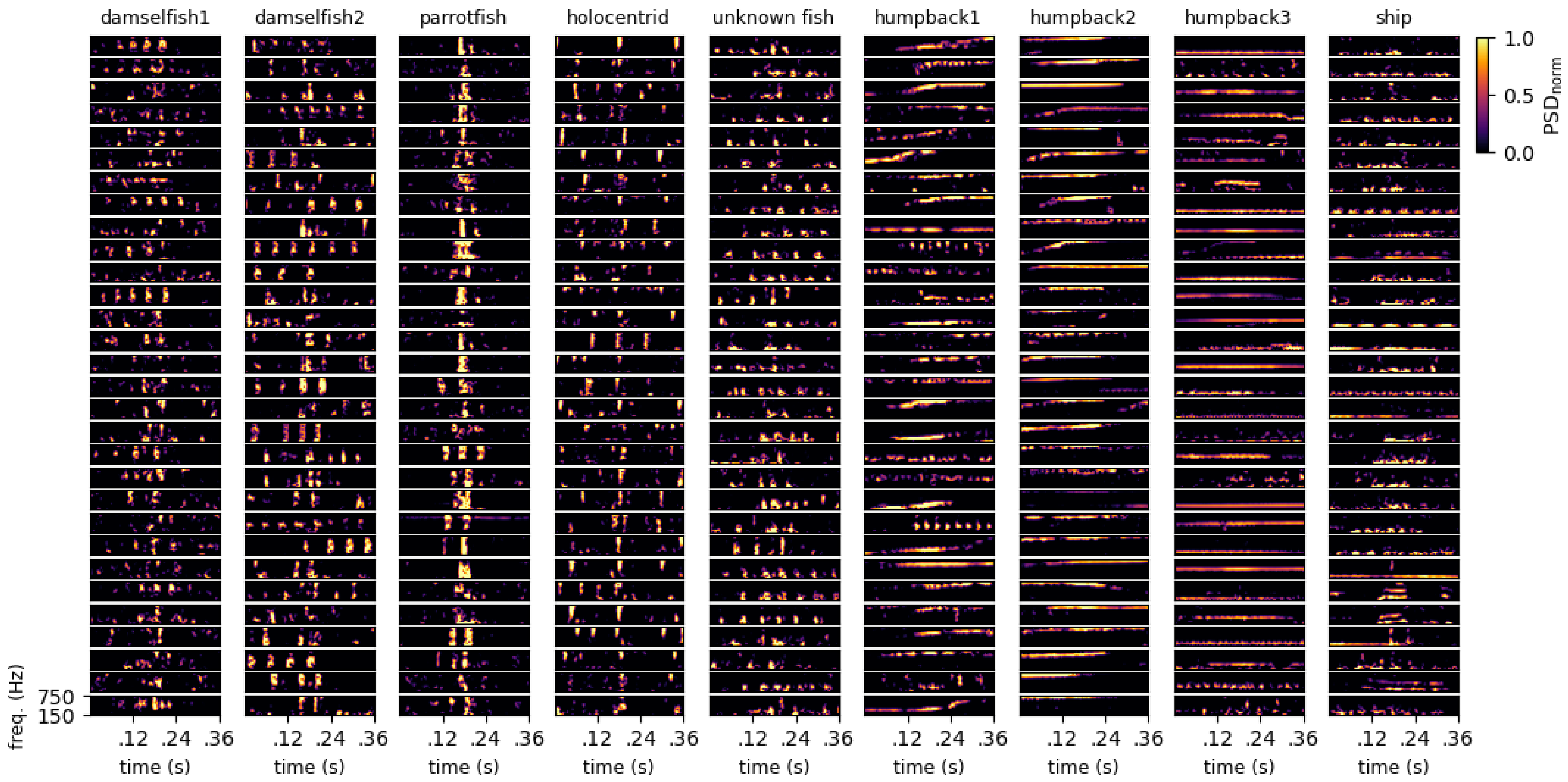

Figure S1B: Recreation of Figure 4 with spectrograms for randomly-selected sample detections in each cluster with likelihood scores greater than 0.5.

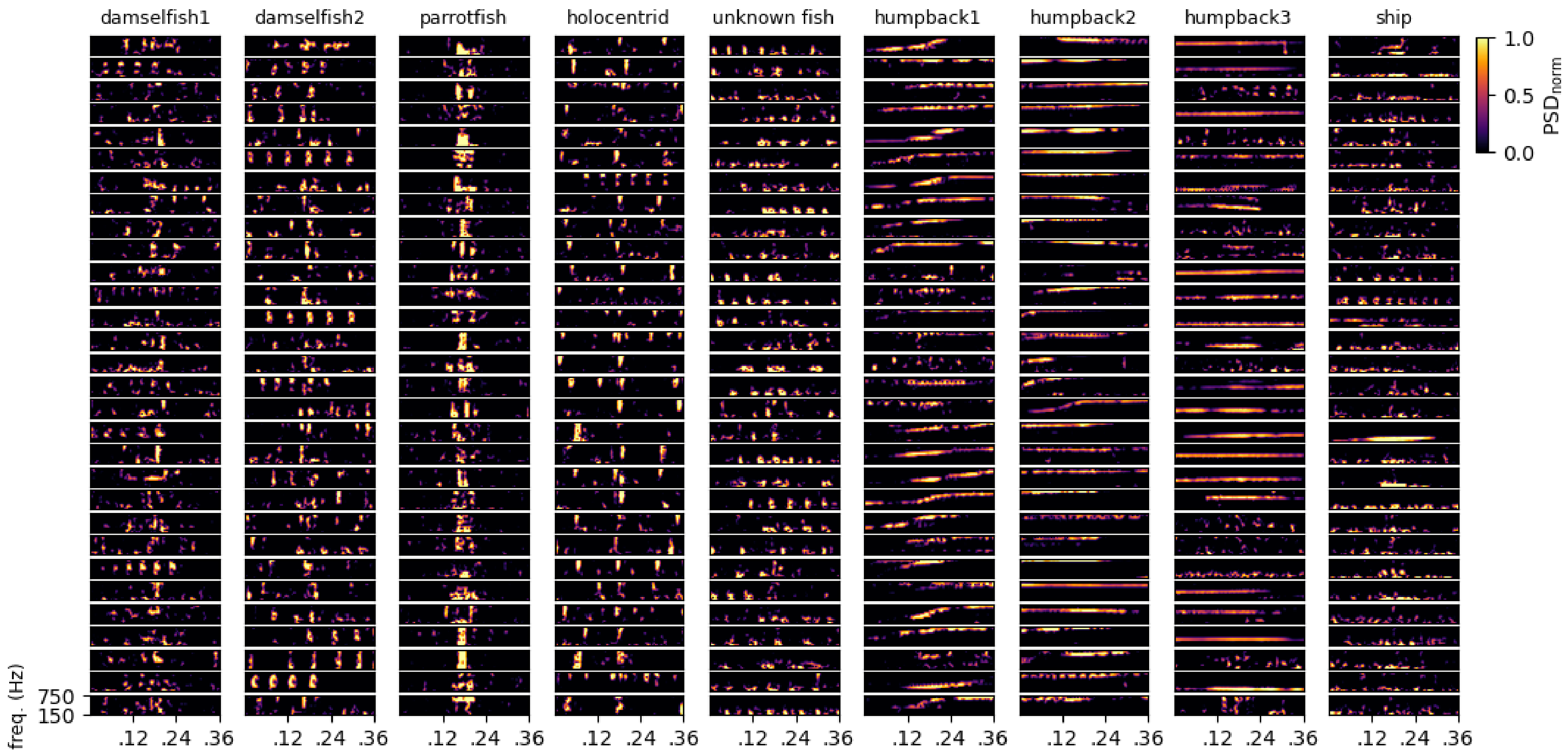

Figure S1C: Recreation of Figure 4 with spectrograms for randomly-selected sample detections in each cluster with likelihood scores greater than 0.25.

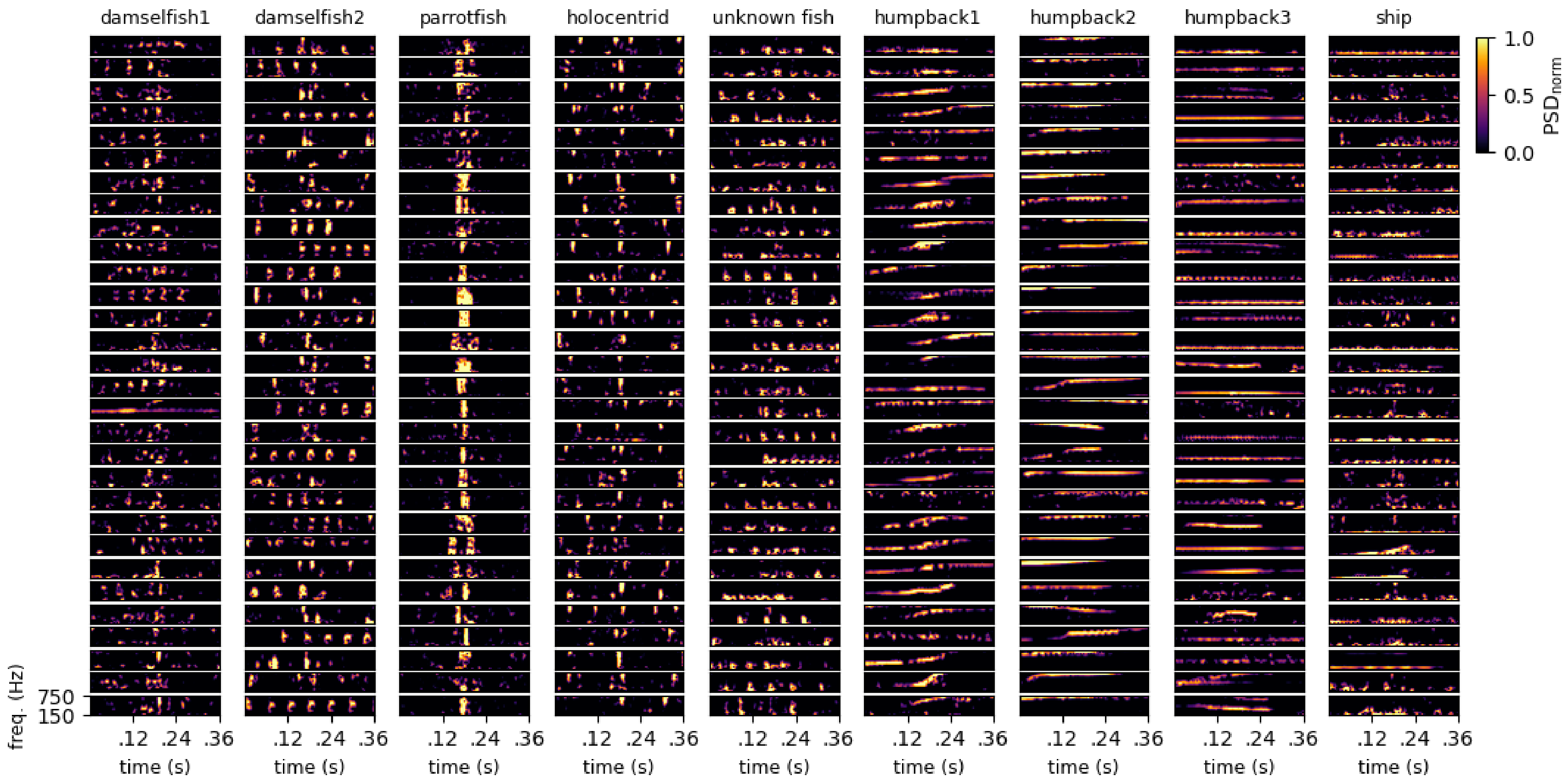

Figure S1D: Recreation of Figure 4 with spectrograms for randomly-selected sample detections in each cluster with likelihood scores greater than 0.
