# Supplementary material for: Multi-class, unsupervised detection and classification of biological and anthropogenic sounds in coral reefs
Source: PLoS Comput Biol. 2026 Jul 20;22(7):e1014516. doi: 10.1371/journal.pcbi.1014516 (PMC13411937; doi:10.1371/journal.pcbi.1014516)
Supplement: S8 Fig — For a sample to be included in the visualization for a given class, it must belong to that cluster and exceed a likelihood threshold L. The thresholds shown are L>0.9 (A), L>0.5 (B), L>0.25 (C), L>0 (D). The diel, lunar, and seasonal trends seen in Fig 7 are mostly preserved as the threshold is lowered. The primary exception is the “ship” class, where a significantly lowered threshold results in elevated nighttime detections, including sunset activity likely associated with biological sound (possible confusion with the “unknown fish” sound). (PDF) [file pcbi.1014516.s009.pdf]

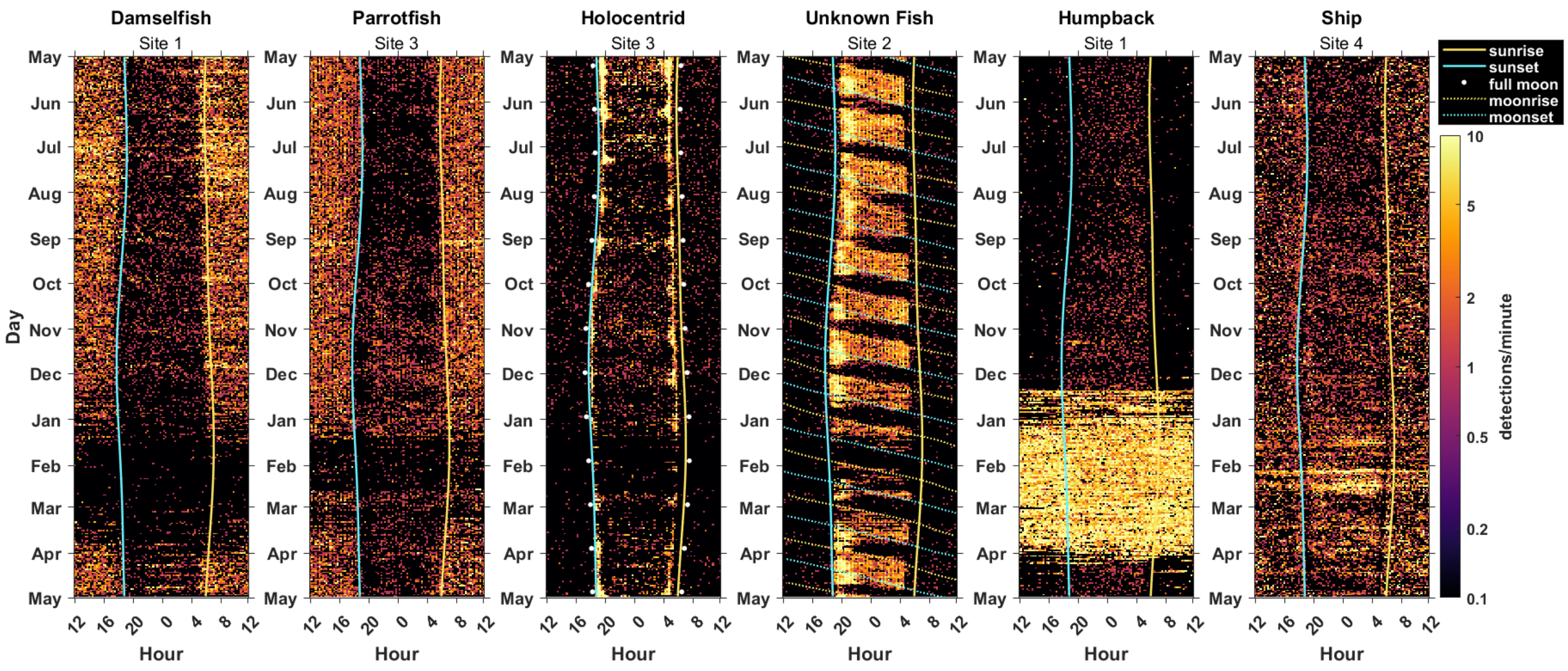

Figure S6A: Recreation of Figure 7 for detections in each cluster with likelihood scores greater than 0.9.

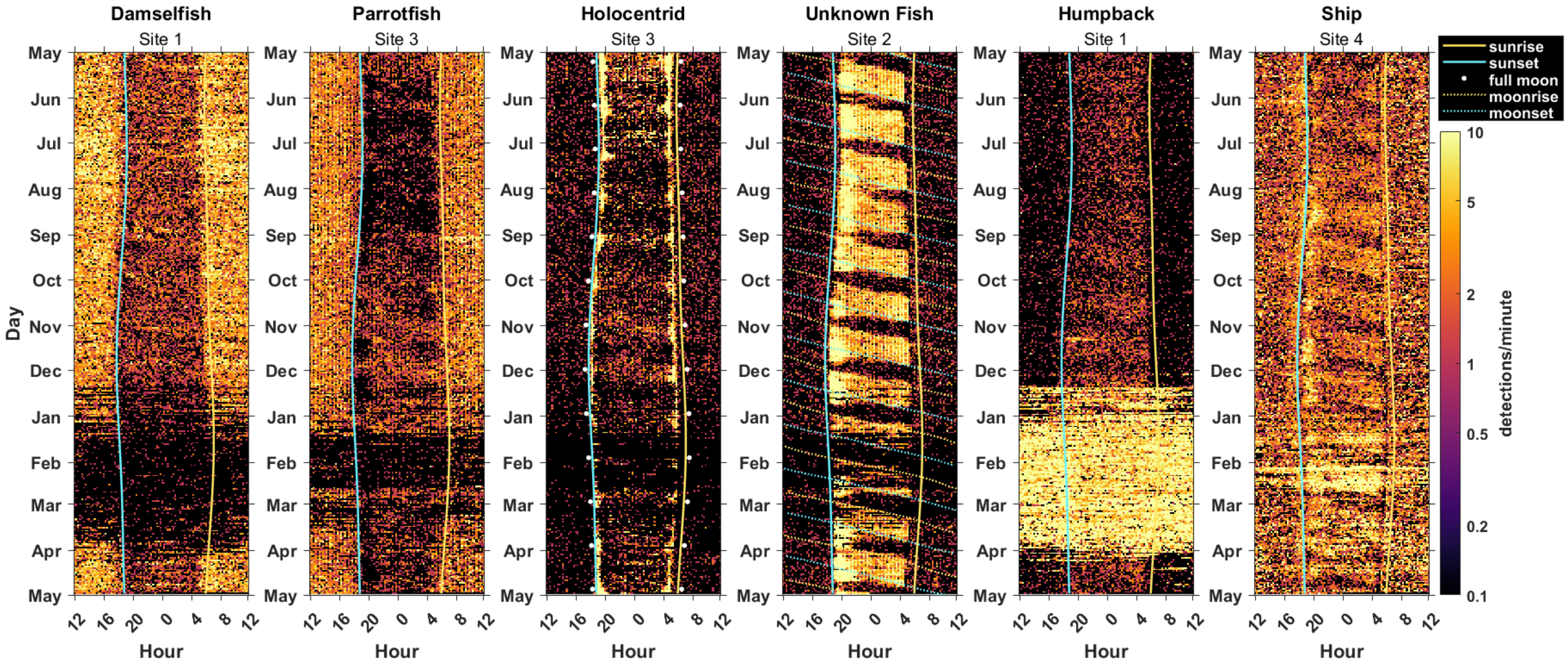

Figure S6B: Recreation of Figure 7 for detections in each cluster with likelihood scores greater than 0.5.

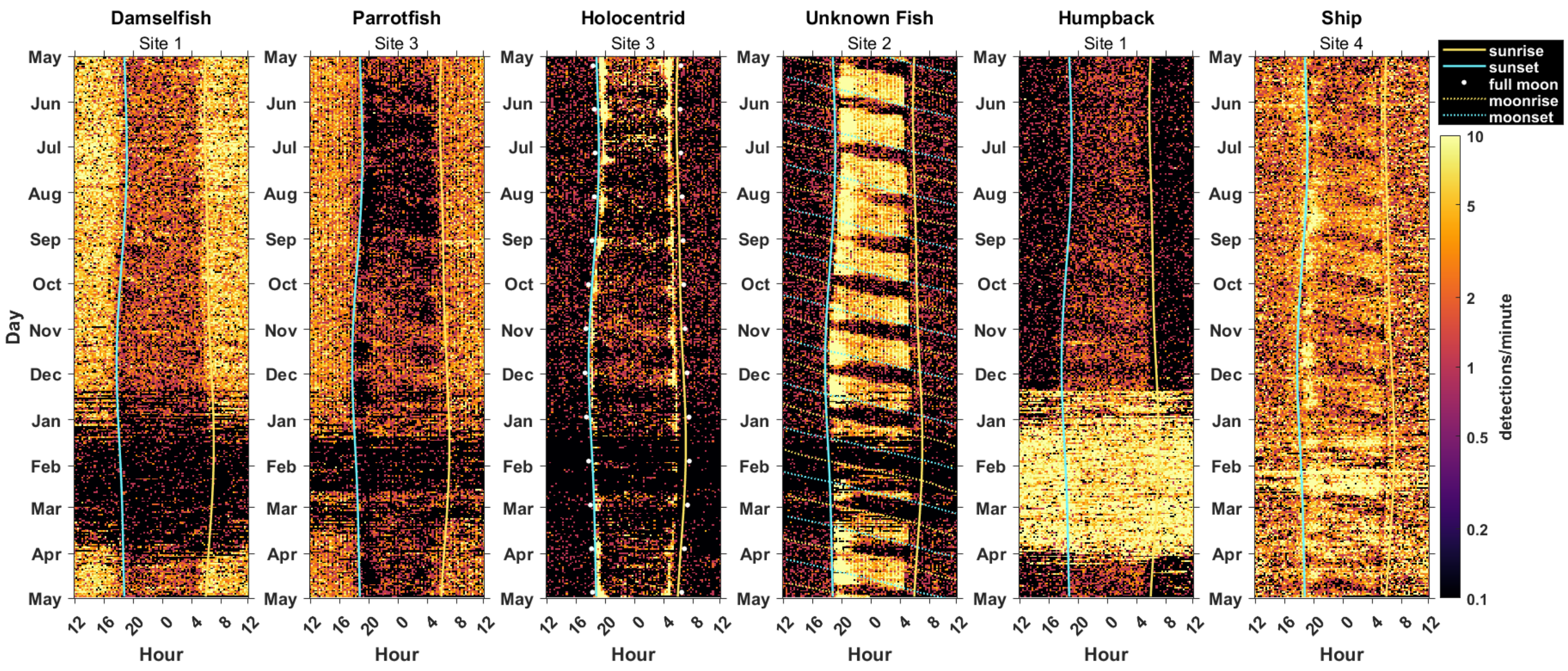

Figure S6C: Recreation of Figure 7 for detections in each cluster with likelihood scores greater than 0.25.

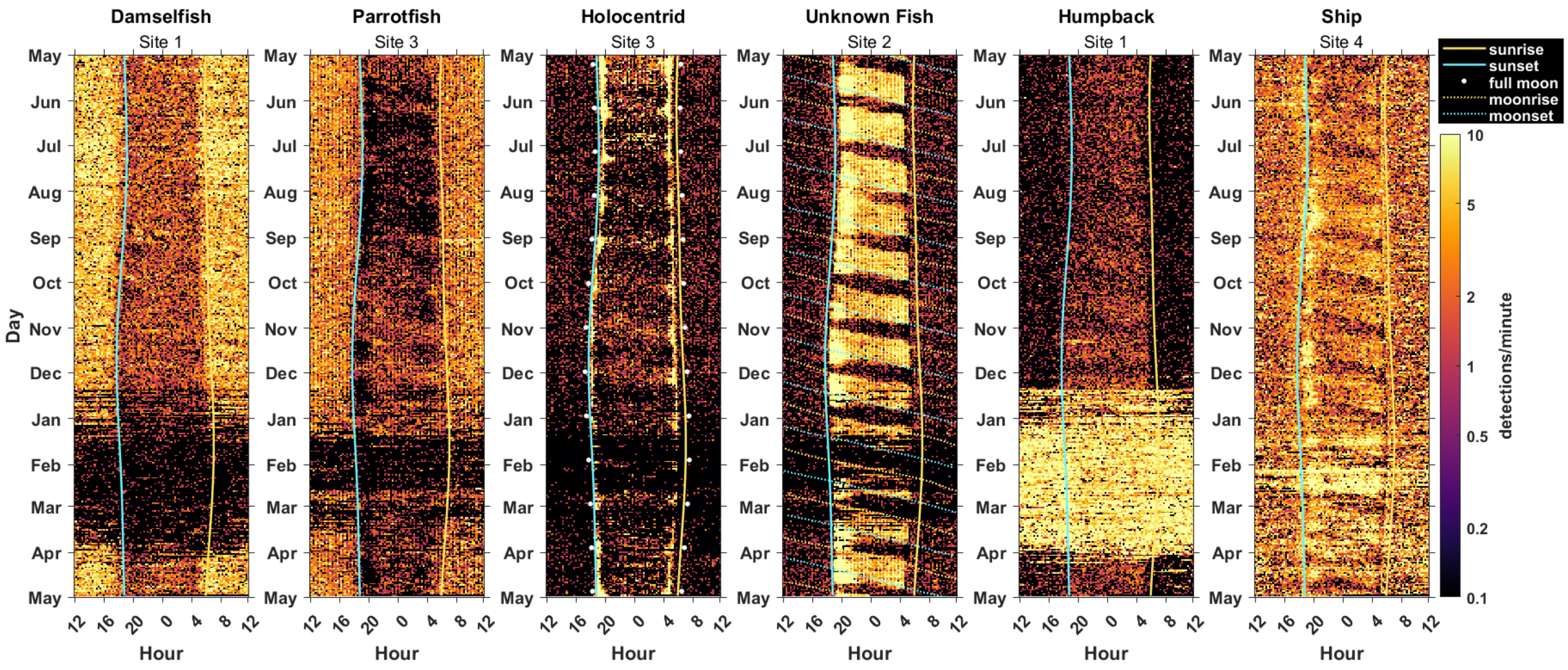

Figure S6D: Recreation of Figure 7 for detections in each cluster with likelihood scores greater than 0.
